# SIRT2-PFKP interaction dysregulates phagocytosis in macrophages with acute ethanol-exposure

**DOI:** 10.3389/fimmu.2022.1079962

**Published:** 2023-01-27

**Authors:** Anugraha Gandhirajan, Sanjoy Roychowdhury, Christopher Kibler, Emily Cross, Susamma Abraham, Annett Bellar, Laura E. Nagy, Rachel Greenberg Scheraga, Vidula Vachharajani

**Affiliations:** ^1^ Department of Inflammation and Immunity, Cleveland Clinic Lerner Research Institute, Cleveland, OH, United States; ^2^ Department of Critical Care Medicine, Respiratory Institute, Cleveland Clinic, Cleveland, OH, United States

**Keywords:** ethanol-exposure, phagocytosis, LC3-associated phagocytosis, sepsis, macrophage, Sirtuin 2, glycolysis

## Abstract

Alcohol abuse, reported by 1/8^th^ critically ill patients, is an independent risk factor for death in sepsis. Sepsis kills over 270,000 patients/year in the US. We reported that the ethanol-exposure suppresses innate-immune response, pathogen clearance, and decreases survival in sepsis-mice *via* sirtuin 2 (SIRT2). SIRT2 is an NAD+-dependent histone-deacetylase with anti-inflammatory properties. We hypothesized that in ethanol-exposed macrophages, SIRT2 suppresses phagocytosis and pathogen clearance by regulating glycolysis. Immune cells use glycolysis to fuel increased metabolic and energy demand of phagocytosis. Using ethanol-exposed mouse bone marrow- and human blood monocyte-derived macrophages, we found that SIRT2 mutes glycolysis *via* deacetylating key glycolysis regulating enzyme phosphofructokinase-platelet isoform (PFKP), at mouse lysine 394 (mK394, human: hK395). Acetylation of PFKP at mK394 (hK395) is crucial for PFKP function as a glycolysis regulating enzyme. The PFKP also facilitates phosphorylation and activation of autophagy related protein 4B (Atg4B). Atg4B activates microtubule associated protein 1 light chain-3B (LC3). LC3 is a driver of a subset of phagocytosis, the LC3-associated phagocytosis (LAP), which is crucial for segregation and enhanced clearance of pathogens, in sepsis. We found that in ethanol-exposed cells, the SIRT2-PFKP interaction leads to decreased Atg4B-phosphorylation, decreased LC3 activation, repressed phagocytosis and LAP. Genetic deficiency or pharmacological inhibition of SIRT2 reverse PFKP-deacetylation, suppressed LC3-activation and phagocytosis including LAP, in ethanol-exposed macrophages to improve bacterial clearance and survival in ethanol with sepsis mice.

## Graphical Abstract

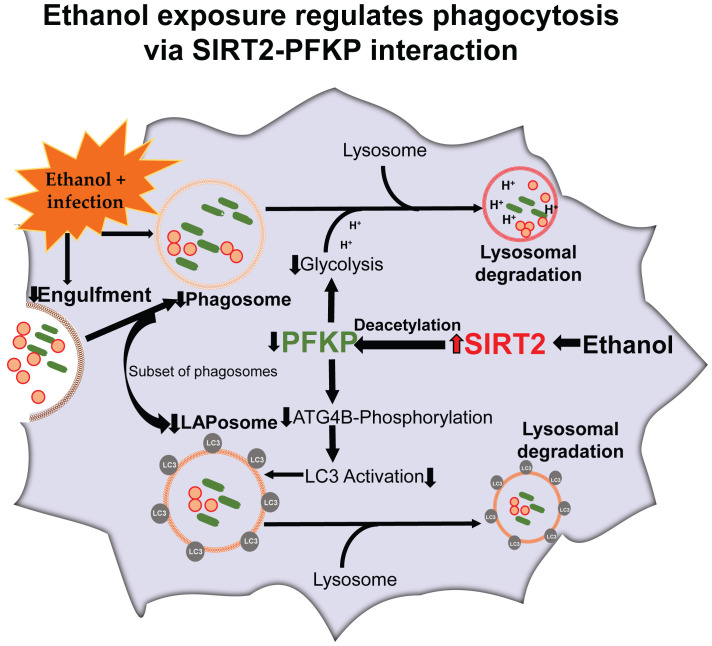

## Introduction

Sepsis, the leading cause of death in hospitalized patients in the US, kills over 270,000 patients/year (1–3). Immune response in sepsis transitions from early/hyper-inflammation to a late/hypo-inflammatory and immunosuppressive phase (4, 5). Majority of sepsis-mortality occurs during immunosuppression (6). Alcohol use disorder, reported by 1/8^th^ critically ill patients, is an independent risk factor for sepsis-mortality (7). Immunosuppression and inability to clear infection with acute ethanol-exposure in sepsis are well described but the molecular targets to reverse immunosuppression have remained elusive (8). We have reported previously, that decreased pathogen clearance and increased mortality in ethanol-drinking mice with sepsis occur *via* increased expression of sirtuin 2 (SIRT2) (9).

Sirtuins (SIRTs) are a highly conserved family of NAD+-dependent histone deacetylases (class III HDAC) that regulate immuno-metabolic responses during acute and chronic inflammatory conditions (10–14). The seven members of SIRT family (SIRTs1-7) disperse throughout cell compartments (15–18). SIRTs 1, 6 and 7 are nuclear, SIRT3, 4 and 5 mitochondrial and SIRT2 is cytoplasmic. While predominantly cytoplasmic, SIRT2 translocates to the nucleus during cellular stress (19). Previously, we have implicated SIRTs in sepsis-induced hypo-inflammation and immunosuppression (20–26). We and others have shown that SIRT2 deacetylates and deactivates NFκB p65 and prolongs the immunosuppressive hypo-inflammatory phase of sepsis with obesity, a common co-morbidity in sepsis patients (22, 27, 28).

Acute ethanol-exposure impairs phagocytosis, which is crucial for pathogen clearance by innate-immune cells including macrophages, during sepsis (29–33). Immune cells use glycolysis to support the high-energy demand during phagocytosis (34–37). The effect of acute ethanol-exposure on glycolysis and glycolysis-regulating enzymes in immunosuppressed macrophages is not fully understood (9, 33, 38). Glycolytic enzymes are known to regulate basic cellular functions and immune response, in addition to the regulation of glycolysis (39–41). Specifically, the platelet isoform of phosphofructokinase (PFKP), a key glycolysis-regulating enzyme, serves as a protein kinase that regulates autophagy. Autophagy is a cytoprotective mechanism by which misfolded proteins, apoptotic cells, cellular debris and pathogens are degraded (42).

PFKP facilitates phosphorylation of autophagy related protein 4B (Atg4B), which activates microtubule associated protein 1 light chain-3B (LC3) to regulate autophagy (43). LC3-associated phagocytosis (LAP) is a subset of phagocytosis that uses parts of autophagy-machinery to enhance segregation and degradation of pathogens and dead-cell debris (44, 45). In LAP, the LC3-II is conjugated to the phagosome membrane to form LAPosome (**Graphical abstract**) (46). Dysregulated LAP is implicated in immunosuppression during sepsis, however the effect of acute ethanol exposure on LAP or LAPosome formation is not well studied (47).

We have reported that ethanol-induced increase in SIRT2 represses immune response and bacterial clearance, while SIRT2 deficiency is associated with increased bacterial clearance and decreased mortality in ethanol with sepsis-mice (25). In the current project, we aimed to elucidate the mechanism by which ethanol-induced SIRT2 regulates the innate immune function of bacterial clearance, phagocytosis, LAP, and cellular metabolism, using mouse-bone marrow- and human blood monocyte- derived macrophages.

## Methods

### Antibodies and reagents

#### Antibodies and reagents used for western blot and immunocytochemistry

PFKP **(**Cell signaling Technology, Danvers, MA, CAT# 8164S), LC3 (Cell signaling Technology, Danvers, MA, CAT# E5Q2K: western blot), LC3 (Novus Biologicals, Centennial, Colorado, CAT# NC100-2220: immunocytochemistry), SIRT2 (Cell signaling Technology, Danvers, MA, CAT# D4050), Atg4B (Cell signaling Technology, Danvers, MA, CAT# 13507S), Rubicon (Cell signaling Technology, Danvers, MA, CAT# 8465S), Beclin-1 (Cell signaling Technology, CAT# 3495S), PFKM (Abcam, Waltham, Boston, USA,CAT#ab154804), VPS34 (Abcam, Waltham, Boston, CAT# ab124905), PFKL (Santa Cruz Biotechnology, Dallas, Texas, USA, CAT# sc-393713), F4/80 (Invitrogen, Rockford, Illinois, USA, CAT# MA1-91124), Anti-rabbit Alexa Fluor 488 (Invitrogen, Rockford, Illinois, USA, CAT# A21206), Anti-rat Alexa Fluor 594 (Invitrogen, Rockford, Illinois, USA, CAT# A21209), Anti-rabbit Alexa Fluor 647 (Invitrogen, Rockford, Illinois, USA, CAT# A21245), Anti rabbit IgG control (Cell signaling Technology, Danvers, MA, CAT# 2729S), anti-mouse IgG, HRP-linked antibody (Cell signaling Technology, Danvers, MA, CAT# 7076), Anti-rabbit IgG, HRP-linked antibody (Cell signaling Technology, CAT# 7074), Acetyl lysine (Novus Biologicals, Centennial, CO, USA, CAT# NB 100-74339) pSerine (Origene, Rockville, MD, USA, CAT# AM00114PU-N), Anti- Turbo GFP (tGFP) antibody (Origene, Rockville, MD, CAT# TA150041), DDK antibody (Origene, Rockville, MD, CAT# TA50011-100), Anti-Ubiquitin antibody (EMD Millipore, Burlington, Massachusetts, USA, CAT#5-944), Biotinylated anti-SIRT2 antibody (R&D system, Minneapolis, MN, USA, CAT# BAF4358), CPA (Novus Biologicals, CAT# NBP1-30993), Beta-actin(Abcam, Waltham, Boston, CAT# ab8226), ECL (Bio-Rad, Hercules, CA, USA, CAT# 1705061). ChromoTek TurboGFP-Trap Magnetic Agarose (Proteintech Group, Inc, Rosemont, IL, CAT# tbtma-10), Magnetic-TUBEs (Life Sensors, Malvern, PA, USA, CAT# UM501M), Zymosan A pHrodo red bio-particles (Invitrogen, Waltham, MA, USA, CAT# P35364), Vybrant phagocytosis assay (Thermo Fisher Scientific, Waltham, MA, USA, CAT# V-6694).

Mouse studies: Cremophor EL (EMD Millipore Corp, Madison, WI, USA, CAT# 238470), AK-7 (Tocris, Minneapolis, MN, USA, CAT# 4754), Lipopolysaccharide (LPS) (Sigma Aldrich, St. Louis, MO, USA, *E. Coli*. O111:B4, CAT# L2630-100 mg), Ethanol (Pharmco by Greenfield global, 200 proof, Brookfield, Connecticut, USA, CAT# 111000200),

#### Glycolysis assay reagents

Glycolysis stress test kit (Agilent technologies, Santa Clara, CA, USA, CAT# 103020-100), glucose-free XF DMEM media (Agilent technologies, Santa Clara, CA, CAT#103575-100) containing 2 mM glutamine (Agilent technologies, Santa Clara, CA, CAT# 103579-100), Hoechst 33342 (Thermo Fisher Scientific, Waltham, MA, USA, CAT# 62249).

#### Immunoprecipitation experiments

Streptavidin magnetic beads (Thermo Fisher Scientific, Waltham, MA, USA, CAT# 88816), Dynabeads protein G magnetic beads (Thermo Fisher Scientific, Waltham, MA, USA, CAT# 10003D).

#### Surface plasmon resonance assay reagents

Recombinant human SIRT2 protein (Sigma-Aldrich, St. Louis, MO, CAT# SRP0116), Human recombinant PFKP protein (His and GST tag), Sino Biological, Wayne, PA, USA, CAT# 15003-H20B), Biacore series S Sensor Chip CM5 (Cytiva, Marlborough, Massachusetts, USA CAT# 29104992), NAD^+^ (nicotinamide adenine dinucleotide, Sigma Aldrich, St. Louis, MO, CAT# 10127973001).

Biologicals for PFKP overexpression in HEK293T cells: mouse wtPFKP (turbo-GFP-tagged) shRNA (Origene, Rockville, MD, CAT# MG210641), KAT5 mouse plasmid (Origene, Rockville, MD, CAT# TR512714), SIRT2 (Myc-DDK-tagged) ShRNA (Origene, Rockville, MD, CAT# MR225715), transfection reagent (Thermo scientific, CAT# R0531), control plasmids (control turbo GFP plasmid pCMV6-AC-GFP (Origene, Rockville, MD, CAT# PS100010), control pCMV6-Entry plasmid (Myc-DDK-tagged; Origene, Rockville, MD, CAT# PS100001), Opti-MEM reduced serum medium (Thermo Fisher Scientific, Waltham, MA, USA, CAT# 31985-062).

#### PFKP plasmid construct and mutagenesis

Mutagenesis was performed on PFKP (turbo-GFP-tagged; Origene, CAT# MG210641: wtPFKP) plasmid at nucleotide residue 1181 from “a” to “g” (a1181g) which change the amino acid from lysine (K) to arginine (R) at 394 residue on PFKP protein sequence (mtPFKP: Origene, Rockville, MD, USA, Ref# CW308061)} by Origene.

#### Tandem Ubiquitin Binding Entities assay reagents

PR-619 (UB/UBI protease inhibitor; Life Sensors CAT# SI9619), 1, 10-phenanthroline (o-PA), (Life Sensors CAT# SI9649).

#### Nucleofection of PFKP siRNA in SIRT2KO-BMDM

PFKP (B) siRNA [(Mouse PFKP siRNA, Origene, Rockville, MD, CAT# SR420114/CAT# SR427511: updated version), nucleofection solution (Lonza Bioscience, Morrisville, NC, USA, Amaxa cell line Nucleofector Kit V, CAT# PS100001, VCA-1003).

#### Human blood isolation

Rapidspheres (STEMCELL technologies, Cambridge, MA, USA, Easy Sep, CAT # 19669), used with magnetic stand (STEMCELL technologies, Easy 50, CAT# 18002). (Macrophage colony-stimulating factor; concentration: 100ug/ml; Murine macrophage colony stimulating factor (M-CSF, Pepro tech, CAT# 315-02-100UG). Gluta MaxTM-1 (Life technology, CAT# 61870-036), Human Serum (Sigma Aldrich, St. Louis, MO, CAT# H4522) penicillin and streptomycin (Life technology, CAT# 15070-063), Fetal bovine serum (FBS, Life technology, Grand Island, NY, USA, CAT # 10082-147) and 1mM EDTA (Ambion, Inc, Austin, TX, USA, CAT # AM9260G), Human M-CSF (Pepro tech, Cranbury, NJ, USA, Cat # 300-25-50Ug).

### Monocyte isolation and macrophage differentiation from healthy volunteer blood samples and *ex vivo* ethanol exposure

All studies were approved by local IRB. Informed consent, as approved by IRB (IRB #19-132), was obtained. Healthy volunteers without active infection and/or immunosuppression/active cancer diagnosis were enrolled and consented for blood draw. Approximately 25 ml blood was collected *via* peripheral vein venipuncture, by a certified phlebotomist and using K2EDTA (BD vacutainer, Franklin Lakes, NJ, USA, CAT#366643, as an anticoagulant) tubes. PBS with 1% FBS and 1mM EDTA was added to the blood to make up a total volume of 50ml. The enriched cell suspension (~25ml) was mixed with Rapidspheres (50µL/ml blood volume). After mixing well, this cell suspension was incubated at room temperature (RT) for 5 minutes. The tube (without lid) was carefully placed into the magnetic stand and incubated at RT for 5 minutes. The enriched cell suspension (~25ml) was transferred into a new tube and again placed into the magnetic stand and incubated at RT for 5 minutes. Final enriched cell suspension containing monocytes was transferred into a new tube and centrifuged at 300 x g for 8 mins at RT. The isolated monocytes were resuspended with human macrophage media RPMI + Gluta Max™-1 along with 10% human serum, penicillin and streptomycin (Life technology, CAT# 15070-063) and the cells were counted. Approximately 2 million monocytes were cultured into a 75cm^2 -^T flask along with Human M-CSF at a ratio of 1:1000 to differentiate into macrophages. The flask was placed at 37 °C for 7 days in the CO_2_ incubator. On the 7^th^ day, the cells were scraped aseptically and exposed to phosphate buffered saline (PBS, vehicle) or ethanol (final concentration, 25 mM) 20h, followed by further stimulation with LPS (final concentration, 100ng/mL) for 4h. Supernatant was collected and cells were lysed subsequently for western blot analysis.

### Mouse studies

The study was approved by the Institutional Animal Care and Use Committee (IACUC) at the Cleveland Clinic Lerner Research Institute (LRI) and experiments were performed according to the NIH guidelines (ACUC approval#: 00002194). The C57BL/6 (wild type: WT) and B6.129-Sirt2tm1.1Fwa/J (global SIRT2 knockout: SIRT2KO) breeding pairs were purchased from the Jackson laboratories (Bar Harbor, ME, USA) and mice were bred in the AAALAC approved animal facility of LRI.

### Bone marrow isolation and *ex vivo* ethanol exposure

Bone marrow derived macrophages were isolated as reported previously (48). Five to six week old male and female mice were used for this study. The bone marrow cells were collected from the femur and tibia of mice and cultured in Roswell Park Memorial Institute (RPMI) (Lonza, Walkersville, MD, USA, CAT#12-702F) containing 10% fetal bovine serum (FBS), 1% penicillin and streptomycin at 37° C and 5% CO_2_ for 6 days along with mouse M-CSF to differentiate into bone marrow-derived macrophages (BMDM). On day 6, the cells were scraped aseptically and exposed to PBS (vehicle) or ethanol (final concentration 25 mM) 20h (49), followed by further stimulation with LPS (final concentration, 100ng/mL) for 4hours. Supernatant was collected and cells were lysed subsequently for western blot analysis. For immunohistochemistry, following ethanol/vehicle exposure and LPS stimulation for 4hours, cells were fixed using 4% paraformaldehyde, washed with PBS and permeabilized with 0.1% Triton-X-100 for 10 minutes. Cells were then washed with PBS, blocked with 2% bovine serum album in PBS for 1 hour, and incubated overnight with respective primary antibody (1:250 dilution in blocking buffer) at 4 °C. Cells were then washed with PBS and incubated in the dark with secondary antibodies for 1 hour at RT. Cells were again washed with PBS and mounted with a DAPI-containing mounting media. Images were acquired using a Leica-confocal microscope using 63X objectives.

### RAW 264.7 cell culture

RAW 264.7 cell macrophages (RAW cells, 1X 10^5^ cells/ml) were cultured in Dulbecco’s modified Eagle’s media (DMEM, Life technologies, CAT# 11995-065) containing 10% FBS, 1% penicillin and streptomycin at 37° C and 5% CO_2_. Cells were stimulated with and without LPS (final concentration, 100ng/mL) for 4hours. Supernatant was collected and cells were lysed subsequently for SIRT2 immunoprecipitation and Western blot analysis.

### Human embryonic kidney (HEK293T) cell culture

HEK293T cells were grown in Minimal Essential media (MEM) supplemented with 10% fetal bovine serum and 1% penicillin and streptomycin at 37° C and 5% CO_2_. Cells were transfected as detailed below in the Transfection method section.

### Immunocytochemistry

Human monocyte- and mouse bone marrow-derived macrophages were exposed to either phosphate buffered saline (PBS: Vehicle) or ethanol (final concentration 25 mM: Ethanol) for 20 hours, followed by further stimulation with LPS (final 100ng/mL) for 4 hours. Cells were fixed using 4% paraformaldehyde and washed with phosphate-buffered saline (PBS). Cells were then again washed with PBS, blocked for 1 hour with 2% BSA containing 0.1% Triton-X-100 and incubated overnight with rabbit anti-SIRT2 or rat anti-F4/80 primary antibody (1:250 dilution in blocking buffer) at 4 °C. Next day, cells were washed three times (duration: 5 minutes) with PBS and incubated in the dark with anti-rabbit or anti-rat secondary antibody for 2 hours at RT. Cells were again washed with PBS and mounted with a DAPI-containing mounting media. Images were acquired using a Leica-confocal microscope using 63X objectives. Images were analyzed using ImagePro Plus software.

### Phagocytosis using Zymosan A pHrodo bio-particles

Cells were exposed to PBS (vehicle) or ethanol (final concentration 25 mM) for 20 hours, followed by further stimulation with LPS (final concentration, 100ng/mL) for 4 hours. Zymosan A pHrodo bioparticles (50 µg/ml/) were added 20 minutes prior to end of the incubation period. At the end of the incubation, cells were fixed with 4% paraformaldehyde for 20 minutes at RT in dark. Cells were then washed with PBS three times and intracellular bio-particles were visualized using a confocal microscope. Zymosan A pHrodo bioparticles stimulate phagocytosis, specifically LAP. Zymosan A bioparticle assay is commonly used to study phagocytosis, including LAP (50–52).

### LC3-associated phagocytosis

LAP was monitored using protocol published in literature (53). Cells were plated on chamber slides exposed to phosphate buffered saline (PBS, vehicle) or ethanol (final concentration 25 mM) for 20 hours, followed by further stimulation with LPS (final 100ng/mL) for 4h. PHrodo bioparticles were added 20 minutes prior to end of the incubation period. At the end of the incubation, fixed with 4% paraformaldehyde for 20 minutes at RT in dark. Cells were then washed with PBS 3 times, blocked for 1 hour, and incubated overnight with LC3 primary antibody at 4°C in dark. The following day, the cells were washed with PBS 3 times and incubated with respective secondary antibody for 2 hours. Cells were again washed with PBS and mounted with a DAPI-containing mounting media. Images were acquired using a Leica-confocal microscope using 63X objectives. Images were analyzed using ImagePro Plus software.

### Phagocytosis using Vybrant phagocytosis assay

The Vybrant phagocytosis assay was carried out per manufacturer’s instruction. Briefly, WT and SIRT2KO BMDM cells were treated with vehicle/ethanol and induced with LPS, as mentioned previously in 96-well microplate. Hereafter, the assay was performed in dark. The fluorescent particles were resuspended completely with concentrated HBSS and sonicated for 2 minutes using water bath sonicator. The suspension was transferred into a clean glass tube containing 4.5 ml of deionized water and sonicated for 2 minutes until all the fluorescent particles are homogeneously dispersed. The media were removed by vacuum aspiration. Fluorescent bio-particle suspension (100 µl/well) was added to all the wells except the unstained control wells. The plate was incubated at 37°C for 2 hours. In the meantime, concentrated trypan blue was resuspended with 4 ml of deionized water in a glass tube and sonicated for 2 minutes. After 2 hours of incubation, the bioparticles were removed by vacuum aspiration, 100 µL of trypan blue suspension was added and incubated for 1 minute. Subsequently, the excess trypan blue was removed by vacuum aspiration. The plate was read on the flex station at RT with the specific wavelength (480, 520, 495) and settings (reading 6 and PMT-auto).

### Glycolysis stress test using Seahorse XF24 analyzer

Extracellular acidification rate (ECAR) was monitored per the manufacturer’s instruction using the glycolysis stress test kit and Seahorse XFe24 Analyzer (Agilent technologies, Santa Clara, CA, USA). BMDM or RAW 264.7 (80000 cells/well) were plated on the XFe24 Seahorse plate were exposed to PBS (vehicle) or ethanol (final concentration 25 mM) for 20h, followed by further stimulation with LPS (final 100ng/mL). At the end of the incubation, cells were washed with the glucose-free XF DMEM media containing 2 mM glutamine and incubated in that media at a non-CO_2_ incubator at 37^°^C for 1 hr. The glycolysis stress test was performed and analyzed according to the manufacturer’s protocol. At the end of the experiment, the cells were stained with Hoechst 33342 and counted using Cytation 5 analyzer. The extracellular acidification rate was presented as ECAR (mpH/minute/10000 cells).

### Lactate assay

Lactate assay kit (Cell Biolabs, Cat. No. MET 5013, San Diego, CA) was used to assess intracellular lactate levels. In brief, WT, SIRT2KO or AK-7-treated WT-BMDM cells were exposed to vehicle/ethanol and induced with LPS, as mentioned previously in 6-well microplates. At the end of the incubation, cells were counted using an automated cell counter and lysed by sonication in assay buffer (100 µl per 10^5^ cells). Cells were then centrifuged at 500g for 5 minutes and supernatants were used for lactate assay as manufacturer’s instruction. Values were normalized to 10^5^ cells.

### Western blot analysis

Whole-cell lysates and immunoprecipitated samples were subjected to SDS–PAGE (4–15% gel) followed by transfer into 0.2 µm pore size polyvinylidene fluoride (PVDF) membrane. Each lane was loaded with 40 µg of protein, in all the gels. The membrane was blocked with 5% skimmed milk in Tris-buffered saline TBS with tween (0.05%) (TBST) for 90 minutes at RT. Blots were incubated with primary antibodies to analyze the expression of proteins overnight at 4°C. The blots were washed three times with TBST and incubated with anti-rabbit/mouse IgG, HRP-linked secondary antibody for 1hour at RT. ECL was used for detection, and images were captured using the ChemiDoc imaging system (Bio-Rad, Hercules, CA, USA) and were quantified using Image J software (NIH, Bethesda, MD, USA).

### Immunoprecipitation

For SIRT2 or Atg4B immunoprecipitation, BMDMs were treated with vehicle/ethanol and stimulated with LPS, as mentioned previously. Post-treatment, the cells were washed with PBS and resuspended in NP-40 lysis buffer (containing 1% Nonidet *P*-40, 50 mM Tris (pH 8), 150 mM NaCl) along with phosphatase and protease inhibitor mixture. Cells were then lysed using a sonicator for 5 × 10 seconds with a 20 second interval between each sonication, at a frequency of 10 kHz. Cell debris was removed by centrifugation at 10,000× *g* for 15 minutes, and the supernatant was treated with biotinylated anti-SIRT2 antibody or with Atg4B antibody as indicated. Respective IgG control antibodies served as negative controls. SIRT2 and biotinylated antibody complexes were immunoprecipitated using streptavidin magnetic beads. Similarly, Atg4B and the antibody complexes were immunoprecipitated using dynabeads protein G magnetic beads. The beads were extensively washed 3 times with washing buffer. After the final wash, the supernatant was discarded, and the pellet was resuspended in 100 µL of 2x sample buffer with BME and denatured at 95°C for 10 minutes. Denatured precipitates were subjected to SDS–PAGE (12% gel) followed by transfer to 0.2 μM PVDF membrane.

### Surface plasma resonance study

The surface plasmon resonance (SPR) experiments were performed in a Biacore model S200 equipment (Cytiva) to analyze the interaction between SIRT2 and PFKP. Recombinant human SIRT2 protein was immobilized on a sensor chip and different concentrations of human recombinant PFKP protein were flowed. Briefly, for the immobilization of human SIRT2, Biacore series S Sensor Chip CM5, which has carboxylic groups available for covalent coupling was used. The carboxylic groups present on the carboxyl methylated 5 (CM5) sensor chip surface were activated with a mixer of EDC (n-ethyl-n-[dimethylaminopropyl] carbodiimide) and NHS (N-hydroxysuccinimide) to form the N-Hydroxysuccinimide (NHS) ester reactive intermediate, after washing with HBS-P+ buffer (0.1 M HEPES, 1.5 M NaCl, 0.5% v/v surfactant P20, pH 7.4), will react with amines dissolved in the immobilization solution. The human SIRT2 ligand (1 μg) was dissolved in 10 mM sodium acetate, pH 4.0 with 20 μM NAD^+^, which was manually immobilized onto the Biacore sensor chip as manufacturer´s instructions. To detect interactions between SIRT2 and PFKP, the human PFKP protein at different concentrations were injected onto the chip where SIRT2 was immobilized. PFKP protein samples were injected serially from lowest to highest concentration (15.62, 31.25, 62.5, 125, 250, 500 and 1000 nM) and a contact time of 120 seconds, a 15 μL/minute flow rate. The surface was regenerated between samples by injecting running buffer (HBS-P+) (600 seconds, 30 μL/minute). The data was quantified using BIAevaluation software S200.

### PFKP over expression in HEK293T cells by transient transfection

Human embryonic kidney (HEK293T) cells were transfected with either mouse wtPFKP or mtPFKP (turbo-GFP-tagged) shRNA to over express PFKP and KAT5 mouse plasmid in the presence or absence of mouse SIRT2 (Myc-DDK-tagged) ShRNA plasmid, using Turbofect as transfection reagent. The control plasmids (control turbo GFP plasmid pCMV6-AC-GFP, control pCMV6-Entry plasmid (Myc-DDK-tagged) were transfected as transfection control. Briefly, on the day before transfection, the cells (density: 1-3 x 10^5^) were plated on 6-well plates in complete medium in order to obtain 50-70% confluence for the day of the experiment. The next day, before transfection, 3 μg of DNA was diluted in 250 μL of Opti-MEM in reduced serum medium. TurboFectin reagent (9 μL; 1: 3 ratio of DNA: transfecting agent) was added to the diluted DNA and gently mixed with the pipet then incubated for 20 minutes at RT. The mixture was added dropwise to the cells while gently shaking the plate to distribute the complex evenly then incubated at 37°C for 24 hours.

### PFKP plasmid construct and mutagenesis

A single site-directed mutagenesis was performed on PFKP (turbo-GFP-tagged wtPFKP) plasmid at nucleotide residue 1181 from adenine to guanine (a1181g) which changes the amino acid from lysine (K) to arginine (R) at 394 residue on PFKP protein sequence mtPFKP (Origene, Rockville, MD, Ref# CW308061) by Origene.

### PFKP over expression and immunoprecipitation using TurboGFP-Trap in HEK293T cells

HEK293T cells were transfected with non-mutated mouse wtPFKP and mtPFKP plasmids in the presence or absence of mouse SIRT2 plasmid along with KAT5 mouse plasmid as described previously. After transfection, cells were resuspended in NP-40 lysis buffer and lysed using a sonicator. Cell debris was removed by centrifugation at 10,000× *g* for 15 minutes, and the supernatant was used for TurboGFP immunoprecipitation using TurboGFP-Trap magnetic agarose beads. The immunoprecipitation protocol was followed per manufacturer’s instructions. Briefly, 25 μL of bead slurry was transferred into a 1.5 ml tube and equilibrated the beads 3 times with 500 μL of dilution buffer. Approximately, 500 μg/ml lysate was added into the equilibrated beads. After overnight incubation at 4°C, beads were washed 3 times with washing buffer. After the final wash, the supernatant was discarded, and the pellet was resuspended in 100 µL of 2x SDS sample buffer with BME and denatured at 95°C for 10 minutes. Denatured precipitates were subjected to SDS–PAGE (12% gel) followed by transfer to 0.2 μM PVDF membrane.

### PFKP over expression and immunoprecipitation of poly-ubiquitinated proteins using magnetic tandem ubiquitin binding entities in HEK293T cells

HEK293T cells were transfected with mouse wtPFKP and mtPFKP plasmids in the presence or absence of mouse SIRT2 plasmid along with KAT5 mouse plasmid as described previously. During 24 hours of transfection 10 μM MG-132 was added to the cells for 4 hours before harvest. After transfection, the cells were resuspended in TUBE lysis buffer (50mM Tris-HCl, pH 7.5, 0.15M NaCl, 1mM EDTA, 1% NP-40, 10% glycerol) with the protease, phosphatase, PR-619 (50µM final concentration, UB/UBI protease inhibitor) and lysed using a sonicator. Also 1, 10-phenanthroline (o-PA), a metal chelator which is a potent inhibitor of metalloproteases was added in the lysis buffer to prevent poly-ubiquitinated protein degradation during cell lysis. Cell debris was removed by centrifugation at 10000X *g* for 15 minutes, and the supernatant was used for immunoprecipitation using Magnetic-TUBEs. The immunoprecipitation was performed per manufacturer’s instructions. Briefly, 100 μL of bead slurry was transferred into a 1.5 ml tube and equilibrated the beads 3 times with 1 ml of TBST wash buffer (20mM Tris-HCl, pH 8.0, 0.15M NaCl, 0.1% Tween-20 with inhibitors). Approximately, 1 mg of total protein containing lysate was added into the equilibrated beads. After 2 hours incubation at 4°C, beads were extensively washed 3 times with 1 ml of washing buffer. After the final wash, the supernatant was discarded, and the pellet was resuspended in 50 µL of 2x SDS sample buffer with BME and denatured at 95°C for 10 minutes. Denatured precipitates were subjected to SDS–PAGE (12% gel) followed by transfer to 0.2 μM PVDF membrane.

### PFKP over expression in RAW 264.7 cell macrophages

RAW 264.7 cell macrophages were transfected with mouse wtPFKP and mtPFKP plasmids in the presence or absence of mouse SIRT2 plasmid along with KAT5 mouse plasmid as described previously. Cells were exposed with ethanol and stimulated with and without LPS (final concentration 100ng/mL) for 4hours simultaneously. Supernatant were collected and cells were lysed subsequently for western blot analysis.

### Nucleofection of PFKP siRNA in SIRT2KO-BMDM

For silencing PFKP, nucleofection was performed using mouse PFKP siRNA in SIRT2KO-BMDM. Cells were removed from petri plates by scraping into 50ml tube and were spun at 335xg for 7 minutes. The supernatant was aspirated completely without any residual media from the cell pellet. Approximately 20uM (0.5 µl) of mouse PFKP (B) siRNA was added to cell pellet (each nucleofection required 15 x10^6^cells/tube) and resuspended the cells in 100 µl Nucleofection solution. The cell suspension was transferred to cuvette and inserted the cuvette in nucleofector cuvette holder to run the nucleofector program in D-032 instrument. The cells were transferred to pre-warmed media after completing the program, and plated in to 100mm tissue culture plate for 24 hours.

### Ethanol exposure in mice

Male and Female C57BL/6 mice (6 weeks old) were housed in standard animal care facilities (5 mice per cage). Age- and gender-matched mice (50% male and 50% female) were randomized and allowed free access to ethanol or water-containing bottles. Mice were exposed to increasing dose of ethanol *via* drinking water; 5% ethanol vol/vol 2 days, followed by 10% ethanol vol/vol for 2 days followed by 30% ethanol vol/vol for 7 days, as described in our previous study(9). On day 11, mice were subjected to experimental conditions as indicated.

### Cecal slurry injection model of sepsis and survival study with AK-7

We studied 7-day survival in ethanol-fed wild type mice with SIRT2 inhibitor AK-7 using cecal slurry (CS) model of sepsis as described previously(9). Mice were given either a single intraperitoneal dose of AK-7 (40mg/Kg body weight; prepared in 25% Cremophor in PBS) (54) or vehicle (Dimethyl sulfoxide, DMSO in 25% Cremophor in PBS), up to 15 minutes prior to CS injection. All mice were injected with CS and received subcutaneous Meropenem (25mg/Kg body weight) twice daily for five doses, starting 18 hours post-CS. Mice were monitored at least twice a day. Pain and distress monitoring, and timely euthanasia as a humane end point to decrease pain and distress, were followed using scoring system as published (22).

### Peritoneal lavage colony forming unit

Bacterial colony forming unit (CFU) was measured in the peritoneal lavage fluid of ethanol-fed AK-7/DMSO-treated mice. Briefly, the ethanol-fed wild-type mice received intraperitoneal injection of either AK-7 or DMSO (vehicle), followed by induction of sepsis using cecal slurry injection. After completion of 7-day survival study (above), the mice were euthanized using cervical dislocation under anesthesia (1–3% Isoflurane- O2 mixture *via* nose cone) on day 8. The anterior abdominal wall was cleaned using 70% ethanol solution. Using aseptic precautions, sterile PBS (3 ml) was injected intraperitoneally, allowed to circulate in the peritoneal cavity and cautiously aspirated using glass pipette with bulb, to perform peritoneal lavage. This fluid, transferred to sterile tubes was used to quantify peritoneal bacterial CFU. Serially diluted peritoneal lavage fluid was plated on LB agar plates using aseptic precautions and incubated overnight at 37°C. The number of aerobic bacterial colonies were counted and expressed as CFU. CFU was calculated using the following formula. CFU/ml = (Number of colonies*dilution factor)/volume of culture plated.

### Image analysis

Signal intensities of Western blot images were quantified by densitometry using Image J software (NIH, Bethesda, MD) and values were normalized with respective loading controls as indicated in the figure. All immunofluorescent images were semi-quantified using ImagePro Plus software (Media Cybernetics, Bethesda, MD) and values were normalized per cell in the field.

### Statistical analysis

All data was expressed as the mean ± standard error of the mean (SEM) with n = 3-4 data point per experimental group, as indicated in figure legends. Statistical analyses was performed using GraphPad Prism software Version 5.02 (GraphPad Software, San Diego, CA, USA). For comparing multiple groups, analysis of variance was used with a NewMann-Koyle *post hoc* test. Mouse survival with AK-7 vs. vehicle (DMSO) treatment was analyzed using Log-Rank test (GraphPad Prism software, as above). Statistical significance was defined as p < 0.05.

## Results

**Figure 1:**
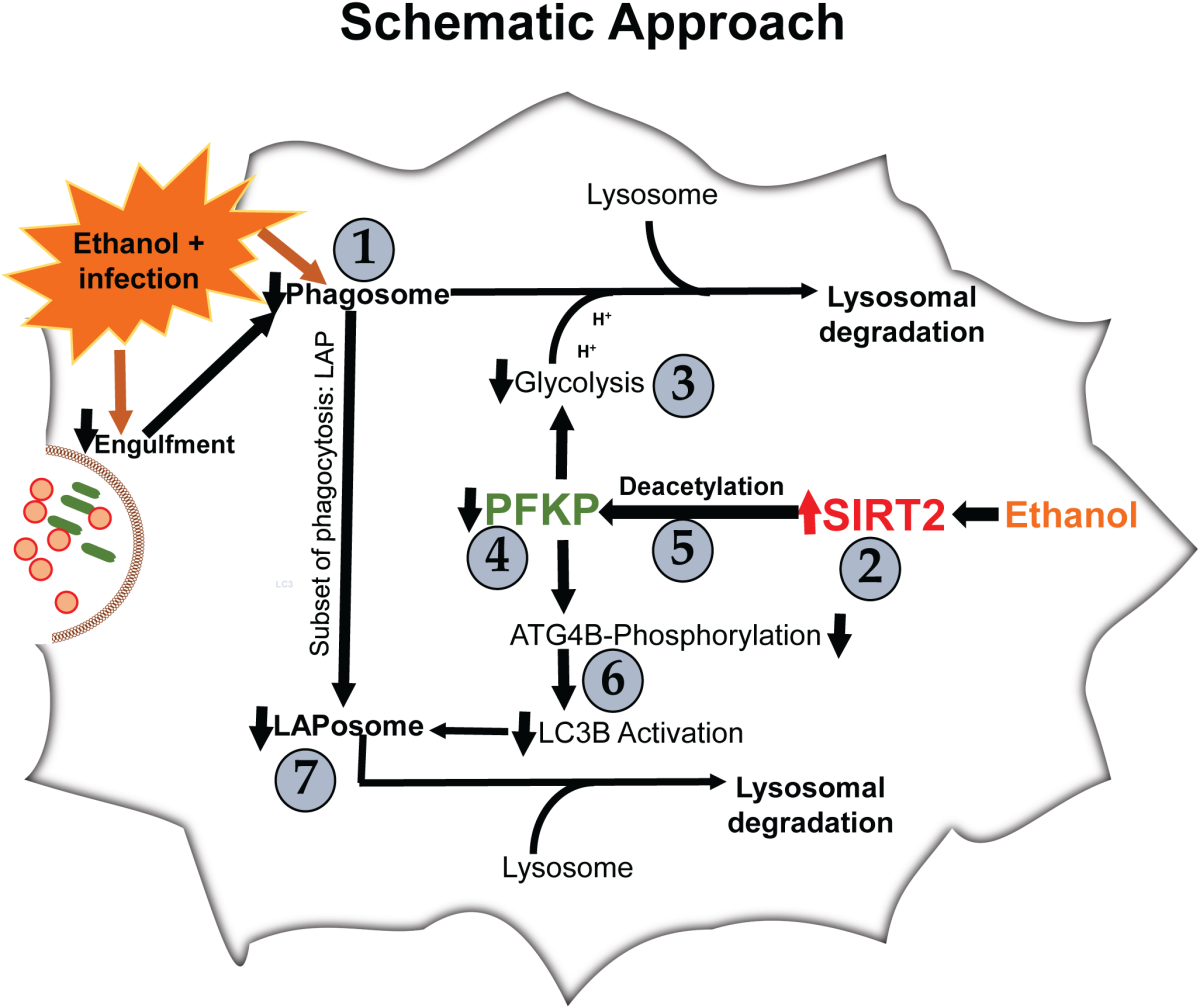
Steps 1-7 describe the order in which results are described.

We examined the effect of acute ethanol-exposure on phagocytosis, glycolysis, glycolytic enzymes and LAP in macrophages in a stepwise manner, as depicted in **Figure 1**.

### Acute ethanol-exposure increases SIRT2 expression and decreases phagocytosis in macrophages

**Figure 2:**
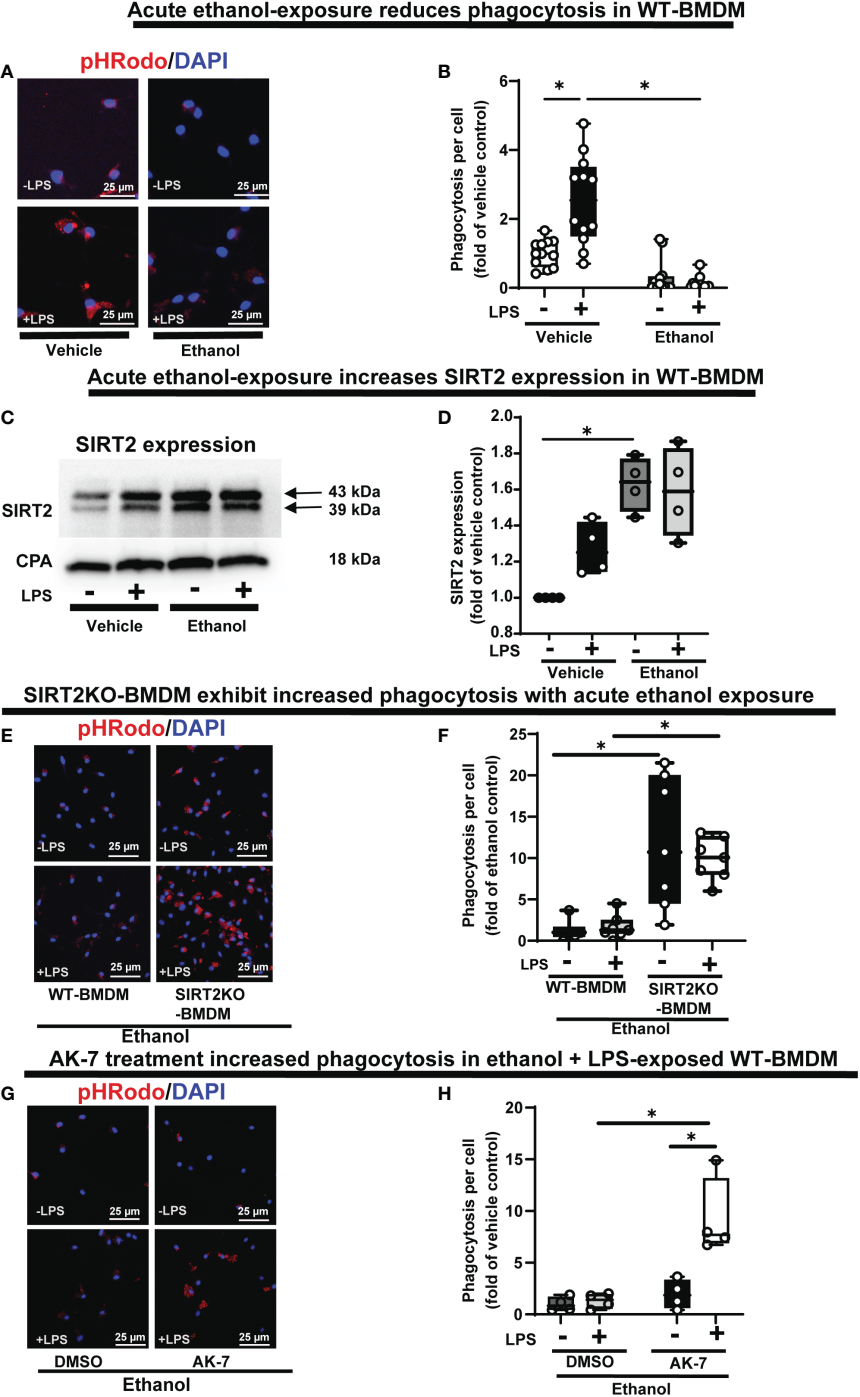
The effect of acute ethanol-exposure on mouse bone marrow derived macrophages (BMDM). Phagocytosis in BMDM exposed to vehicle or ethanol ± LPS to study phagocytosis using pHrodo bioparticles and SIRT2 expression. **(A)** Representative images of intracellular pHrodo bioparticles in vehicle or Ethanol-exposed WT-BMDM ± LPS. **(B)** Fluorescence quantification of pHrodo bioparticles in WT-BMDM (n=4 repetitions/group; * p<0.05). **(C)** SIRT2 protein expression detected by western blot in Vehicle- or Ethanol-exposed BMDM cells ± LPS. **(D)** Western blot image quantification of SIRT2 protein blot in vehicle or ethanol-exposed BMDM cells ± LPS (n = 4 blots/group; * *p* < 0.05). **(E)** Representative images of pHrodo bioparticles in Ethanol-exposed WT-BMDM and SIRT2KO-BMDM ± LPS. **(F)** Fluorescence quantification of pHrodo bioparticles in Ethanol-exposed WT-BMDM, and SIRT2KO-BMDM ± LPS. * p < 0.05. **(G)** Representative images of pHrodo bioparticles in Ethanol-exposed WT-BMDM, co-treated with SIRT2 inhibitor AK-7 or DMSO ± LPS. **(H)** Fluorescence quantification of pHrodo bioparticles in Ethanol-exposed WT-BMDM with AK-7/DMSO ± LPS stimulation. * p < 0.05.

Phagocytosis is critical for bacterial clearance in sepsis. We used BMDM from C57BL/6 (WT) mice with LPS stimulation model of sepsis. Differentiation into macrophages was confirmed by F4/80 expression in over 95% of cells (**Supplementary Figure S1**) (55, 56). To examine the effect of acute ethanol-exposure (duration: 24h) (38, 57) on WT-BMDM-phagocytosis, we used Zymosan A pHrodo bioparticles (Step 1: **Figure 1**). Zymosan A bioparticles are known to stimulate phagocytosis (50–52). As expected, the Vehicle-exposed WT-BMDM showed a robust increase in phagocytosis with LPS (Vehicle: + LPS/-LPS= 2.5 fold-increase). However, the Ethanol-exposed macrophages showed a profoundly muted phagocytosis (Ethanol: +LPS/-LPS=0.38 fold-change) (representative image: **Figure 2A**, image quantification: **Figure 2B**). The red color in pHrodo assay is pH sensitive and indicates internalization of bioparticle in a phagosome within the cell. To further investigate whether the ethanol-exposure affects the ability of the macrophages to engulf/internalize the bio particles, we used Vybrant assay with fluorescein-labeled *E. coli* bio particles. We observed a significantly decreased capacity to engulf E. coli particles in Ethanol-exposed WT-BMDM with LPS stimulation compared to vehicle-exposure (**Supplementary Figure S2A**). Lactate can suppress pro-inflammatory immune response in macrophages (58). To assess whether increased lactate was responsible for repressed phagocytosis here, we studied lactate concentrations in vehicle and Ethanol-exposed macrophages± LPS. We observed, that the lactate levels were significantly lower in Ethanol-exposed macrophages with LPS (**Supplementary Figure S3A**).

We reported increased SIRT2 expression in acute Ethanol-exposed RAW 264.7 cell macrophages previously (9). Here, using western blot assay, we observed increased SIRT2 expression in ethanol- vs. Vehicle-exposed WT-BMDM without LPS (60% increase in Ethanol-LPS, p<0.05) and with LPS (50% increase in Ethanol+ LPS, p>0.05) vs. control (Vehicle-LPS) (representative image: **Figure 2C** and WB quantification: **Figure 2D**) (Step 2: **Figure 1**), consistent with our previous report (9).

### SIRT2 deficiency preserves phagocytosis and glycolysis in macrophages with acute ethanol-exposure

To further investigate the role of SIRT2 with acute ethanol-exposure, we studied the effect of SIRT2 deficiency on phagocytosis, using genetic and pharmacological approaches. Ethanol-exposed BMDM from genetically deficient SIRT2KO mice (SIRT2KO-BMDM) showed increased phagocytosis vs. WT-BMDM without LPS (SIRT2KO/WT = 9 fold-increase, p<0.05) and with LPS (SIRT2KO/WT = 5 fold-increase, p<0.05) (representative image: **Figure 2E**, image quantification: **Figure 2F**). The engulfment capacity using *E. coli* bioparticles (Vybrant assay) also significantly increased in ethanol exposed SIRT2KO-BMDM vs. WT-BMDM without LPS, and remained high with LPS stimulation (**Supplementary Figure S2B**).

SIRT2 inhibitor AK-7 binds to the NAD+ binding site on SIRT2 leading to a competitive inhibition of SIRT2 (59). Using pharmacological approach, we observed as expected, that acute ethanol-exposure repressed phagocytosis in WT-BMDM with DMSO (vehicle for AK-7)-treatment. In contrast, the AK-7-treated-Ethanol-exposed WT-BMDM exhibited a dramatic increase in phagocytosis with LPS stimulation (representative image: **Figure 2G**, image quantification: **Figure 2H**) (DMSO: +LPS/-LPS= 1.3 fold-increase, p>0.05 vs. AK-7: +LPS/-LPS=4 fold-increase, p<0.05). There was no significant increase in phagocytosis in the AK-7 vs. DMSO-treated cells without LPS. Together, these data demonstrate that increased SIRT2 is associated with repression while SIRT2 deficiency with preserved phagocytic response, in Ethanol-exposed macrophages. Furthermore, we found higher lactate concentrations in Ethanol-exposed SIRT2 deficient BMDM (SIRT2KO-BMDM: **Supplementary Figure S3B** and AK-7 treated WT-BMDM: **Supplementary Figure S3C**) vs. WT-BMDM counterparts.

**Figure 3:**
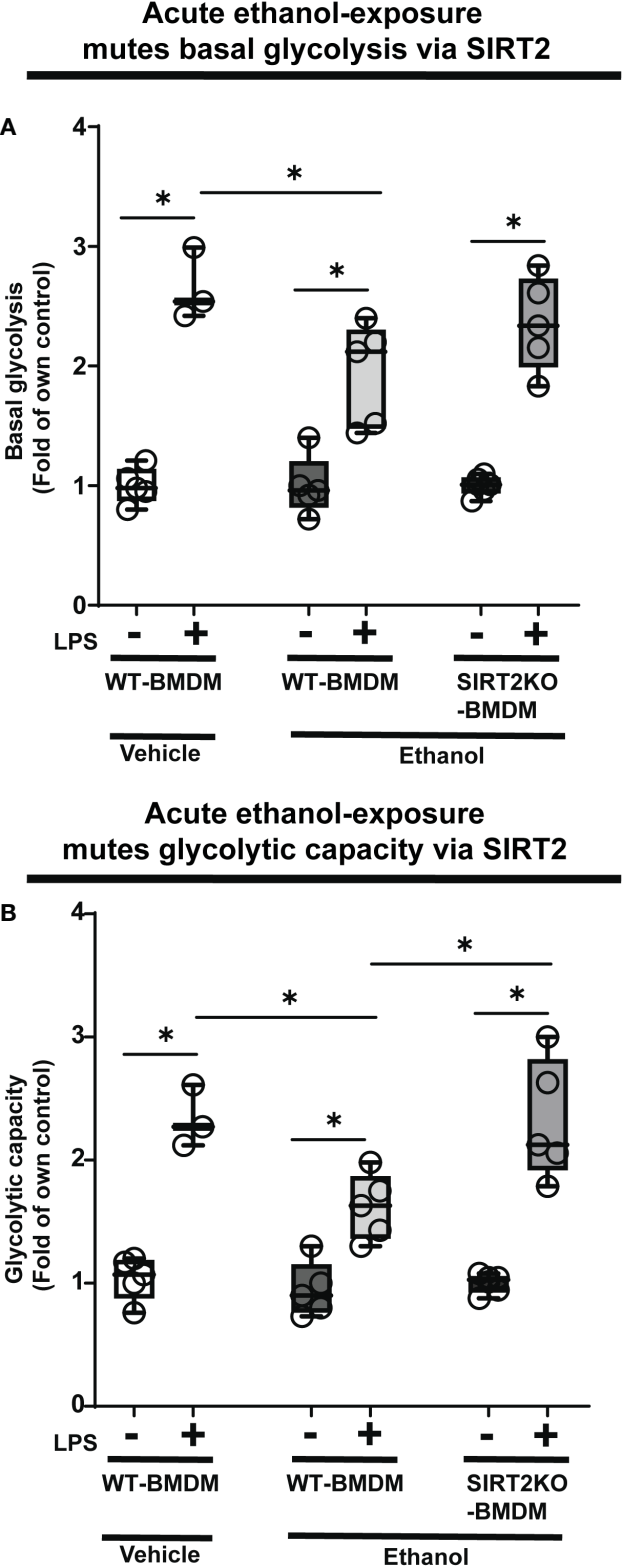
The effect of acute ethanol-exposure on glycolysis in macrophages. Glycolysis assay in BMDM exposed to vehicle or ethanol ± LPS. Y-axis represents response to LPS (+ LPS) as a fold of respective group without LPS (-LPS) represented as “Fold of own control”. **(A)** Basal glycolysis (mean ECAR after glucose addition) in Vehicle-exposed WT-BMDM, Ethanol-exposed WT-BMDM and Ethanol-exposed SIRT2KO-BMDM ± LPS stimulation. **(B)** Glycolytic capacity (mean ECAR upon addition of ATP synthase inhibitor oligomycin) in Vehicle-exposed WT-BMDM, Ethanol-exposed WT-BMDM and Ethanol-exposed SIRT2KO-BMDM ± LPS stimulation (*p<0.05).

Glycolysis is essential to fuel increased energy-demand for phagocytosis (34–37). Hence, we studied the effect of ethanol-exposure on glycolysis (glycolysis stress test: Seahorse XF24) in WT-BMDM (Step 3: **Figure 1**). We observed a muted basal glycolysis in response to LPS in ethanol- vs. Vehicle-exposed WT-BMDM (Vehicle: +LPS/-LPS=2.65 fold-increase vs. Ethanol: +LPS/-LPS= 1.93 fold-increase). However, Ethanol-exposed SIRT2KO-BMDM showed a robust increase in basal glycolysis with LPS (+LPS/-LPS=2.35 fold-increase) (**Figure 3A**).

Similarly, we observed muted glycolytic capacity in response to LPS in ethanol- versus Vehicle-exposed-WT-BMDM (Vehicle: +LPS/-LPS=2.25 vs. Ethanol: +LPS/-LPS=1.11 fold-increase). However, Ethanol-exposed SIRT2KO-BMDM exhibited robust increase in glycolytic capacity in response to LPS (+LPS/-LPS=2.37-fold-increase) (**Figure 3B**).

### Acute ethanol-exposure impairs PFKP expression in macrophages

**Figure 4:**
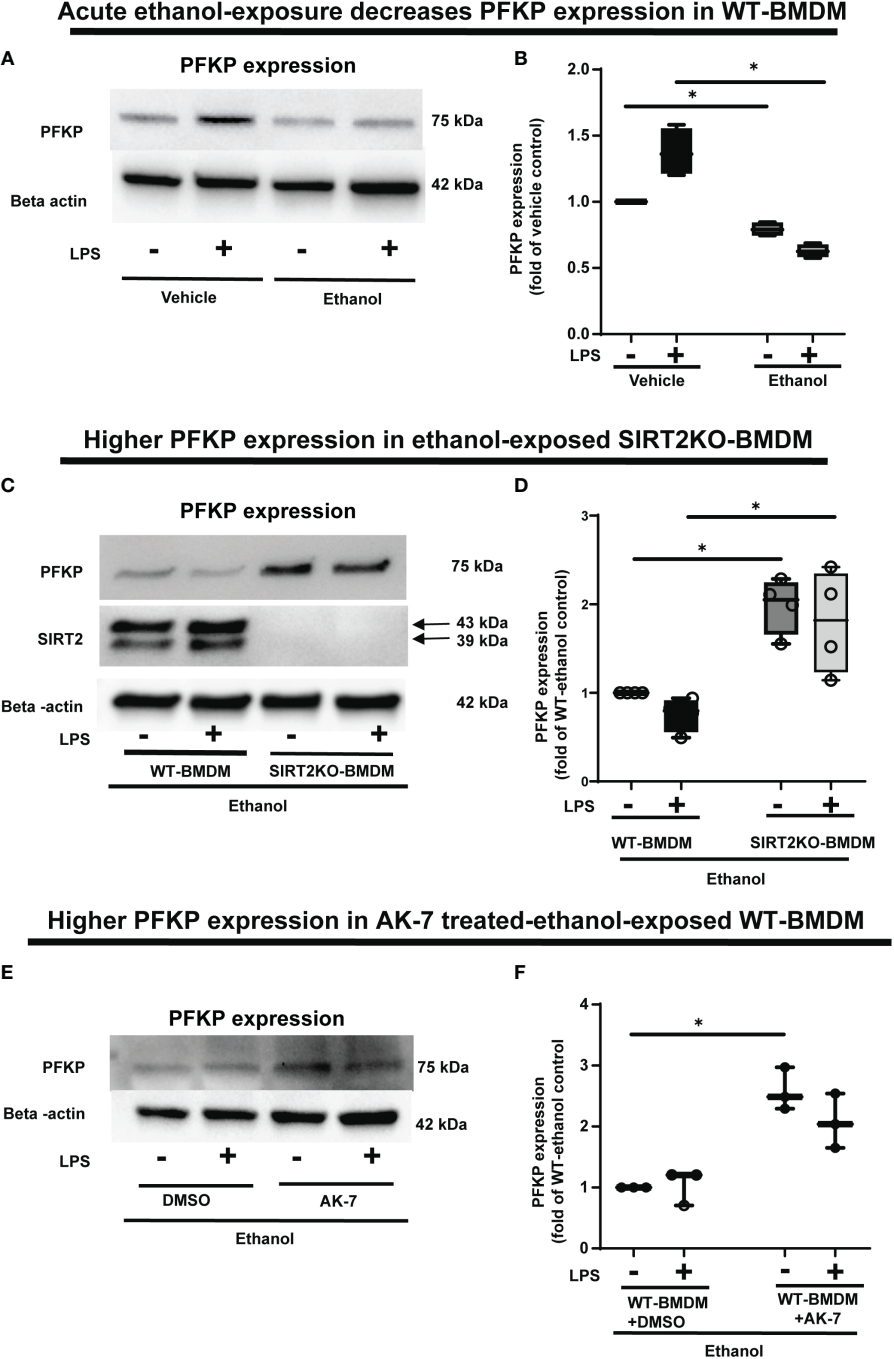
The effect of acute ethanol-exposure-induced SIRT2 on PFKP expression in macrophages. **(A)** PFKP expression in WT-BMDM exposed to vehicle or ethanol ± LPS by western blot analysis. **(B)** PFKP western blot image quantification of PFKP protein in vehicle vs. ethanol exposed WT-BMDM, Y axis represents fold of vehicle-LPS (fold of vehicle control) (n = 4 blots; * *p* < 0.05). **(C)** PFKP expression in Ethanol-exposed WT-BMDM and SIRT2KO-BMDM ± LPS. **(D)** Western blot image quantification of PFKP protein in ethanol exposed WT-BMDM and SIRT2KO-BMDM. Y axis represents fold of Ethanol-exposed WT-LPS (fold of WT- ethanol control) (n = 4 blots; * *p* < 0.05). **(E)** PFKP expression in Ethanol-exposed WT-BMDM treated with AK-7 or DMSO ± LPS. **(F)** Western blot image quantification of PFKP in AK-7 vs. DMSO treated-Ethanol-exposed WT-BMDM ± LPS, Y axis represents fold of Ethanol-exposed WT-LPS (fold of WT- ethanol control) (n = 4 blots; * *p* < 0.05).

To investigate the mechanisms by which ethanol mutes glycolysis, we examined the expressions of key glycolysis-regulating enzyme phosphofructokinase (PFK) in Ethanol-exposed WT-BMDM (Step 4: **Figure 1**). We examined three isoforms of PFK, the platelet isoform (PFKP), liver isoform (PFKL) and muscle isoform (PFKM). We observed that the PFKP expression decreased in ethanol- versus Vehicle-exposed WT-BMDM ± LPS. Specifically, without LPS, we found, that PFKP expression in Ethanol group was 79% of Vehicle, a decrease by 21%. In cells with LPS, the PFKP expression was found to be 62% of the Vehicle, a decrease by 38% (representative WB image **Figure 4A**; WB quantification: **Figure 4B**). The other two isoforms of PFK, PFKL (representative blot: **Supplementary Figure S4A**, WB quantification: **Figure S4B**) or PFKM (representative blot: supplementary **Figure S4C**, WB quantification: **Supplementary Figure S4D**) remained unchanged in ethanol vs. Vehicle-exposed WT-BMDM ± LPS. Similarly, we did not find differential expressions of other important glycolysis regulating enzymes hexokinase I, hexokinase II, pyruvate kinase M1 or pyruvate kinase M2 in ethanol- vs. Vehicle-exposed WT-BMDM ± LPS stimulation (data not shown).

To investigate the role of SIRT2 further, we examined PFKP expression in Ethanol-exposed macrophages with SIRT2 deficiency. Ethanol-exposed SIRT2KO-BMDM exhibited significantly higher PFKP expression vs. WT-BMDM ± LPS. Specifically, in Ethanol-exposed cells without LPS, the PFKP expression increased 1.9-fold in SIRT2KO vs. WT-BMDM-LPS baseline. With LPS stimulation, we found that PFKP expression increased by 2.4 fold in SIRT2KO+LPS vs. WT-BMDM baseline (representative image **Figure 4C**; WB quantification: **Figure 4D**).

Using a pharmacological approach, we found that in Ethanol-exposed WT-BMDM without LPS stimulation, the PFKP expression increased significantly to 2.5 fold in AK-7 vs. DMSO-LPS baseline (p<0.05). Similarly, in ethanol-exposure with LPS, the PFKP expression increased to 2-fold in AK-7 treated cells vs. DMSO-LPS baseline; although a strong trend, this increase was not statistically significant (representative image **Figure 4E**; WB image quantification: **Figure 4F**). We then proceeded to investigate the interaction between SIRT2 and PFKP.

### SIRT2 directly interacts with and deacetylates PFKP

Multiple lysine acetylation and ubiquitination sites for PFKP protein are described in literature (60). SIRTs1 and 2 are known to promote poly-ubiquitination and degradation of proteins through deacetylation (61). Acetylation of PFKP at human lysine 395 (hK395) is critical for its glycolytic function (62). Therefore we tested whether a direct interaction with SIRT2 leads to PFKP-deacetylation at mouse mK394 (hK395), followed by ubiquitination and subsequent proteasomal degradation, in Ethanol-exposed WT-BMDM. Post-translational modification analysis of PFKP protein by PhosphoSitePlus (https://www.phosphosite.org) revealed that mK394 (hK395) residue of PFKP is also a target of ubiquitination, which supports our hypothesis.

**Figure 5:**
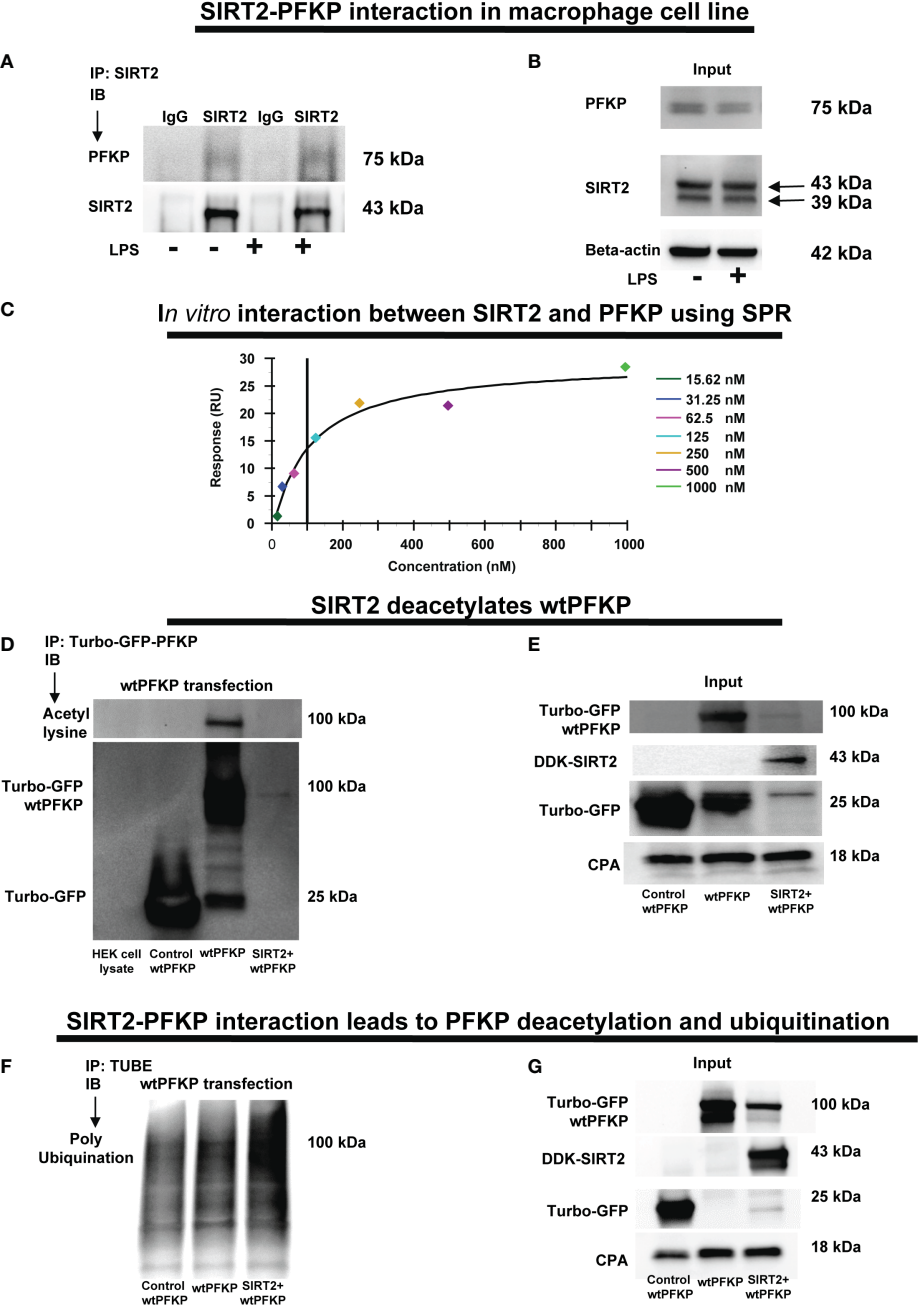
SIRT2-PFKP *in vivo* and *in vitro* interaction. **(A)** RAW264.7 cell macrophages (RAW) ± LPS. IP of whole-cell lysates using an anti-SIRT2 antibody followed by IB analysis of PFKP and SIRT2. IP with isotype IgG control antibody was used as a negative control. **(B)** Western blot analysis of PFKP and SIRT2 in the whole cell lysate used as input for the SIRT2 IP. **(C)** *In-vitro* interaction between SIRT2 and PFKP using SPR. SIRT2 protein immobilized onto sensor chip and the PFKP was flowed at various concentration (15.62, 31.25, 62.5, 125, 250, 500 and 1000nM). The response units on Y axis (RU) represent quantitative assessment of protein-protein interaction. **(D)** wtPFKP and control plasmid transfection and IP, using turbo-GFP-trap in HEK293T cells, in presence or absence of SIRT2 followed by IB analysis of acetyl lysine, turbo-GFP-wtPFKP and control for PFKP (turbo-GFP). HEK293T cell lysate without transfection used as a negative control. **(E)** Western blot analysis of control-PFKP (Turbo-GFP), wtPFKP (Turbo-GFP-wtPFKP), DDK-SIRT2 and CPA in whole cell lysate used as input for the turbo-GFP IP. **(F)** wtPFKP transfection and IP using magnetic-TUBEs in HEK293T cells, in presence or absence of SIRT2 followed by IB analysis of ubiquitination. **(G)** Western blot analysis of turbo-GFP wtPFKP, DDK-SIRT2, turbo-GFP and CPA in whole cell lysate used as an input for the TUBE IP.

We first studied SIRT2-PFKP interaction (Step 4: **Figure 1**) using immunoprecipitation of Vehicle-exposed RAW 264.7 cells. With SIRT2-immunoprecipitation (IP), we observed PFKP co-expression by immunoblot (IB) (representative IP images: **Figure 5A** and respective input blots: **Figure 5B**), indicating SIRT2-PFKP interaction. To further confirm the SIRT2-PFKP interaction *in vitro*, we performed surface plasmon resonance (SPR) assay. SPR results represent steady state equilibrium binding model and demonstrated a direct association between SIRT2 and PFKP proteins. Concentration of PFKP is indicated on X-axis and the Y axis represents response units (RU) as a quantitative assessment of protein-protein (SIRT2-PFKP) interaction (63). The interaction kinetics revealed, that increasing concentrations of PFKP (15.62, 31.25, 62.5, 125, 250, 500 and 1000nM), had increasing binding to affinity for SIRT2 (increasing RU) (**Figure 5C**) with KD (affinity constant) value of 100nM.

SIRT2 is a deacetylating enzyme. To further investigate whether the direct interaction between SIRT2 and PFKP leads to PFKP-deacetylation (Step 5: **Figure 1**), we studied acetylated PFKP expression using co-transfection of PFKP and SIRT2 in HEK293T cells. We used HEK293T cells to allow for stable transfection of both the plasmids. With immunoprecipitation (IP) for PFKP (**Figures 5D, E**), we observed acetylated-PFKP (IB) in cells transfected with PFKP plasmid alone (wild type: wtPFKP). However, in SIRT2+wtPFKP-co-transfected cells, we found significantly less acetylation (deacetylation) of PFKP.

Thus, we found that the SIRT2-PFKP interaction decreased PFKP expression, either in Ethanol-exposed BMDM (**Figures 4A, B**) or SIRT2+wtPFKP co-transfected (SIRT2+wtPFKP) RAW 264.7 cells (Input: **Figure 5E**). Hence, we further investigated whether SIRT2 affects ubiquitination and degradation of PFKP, by co-transfecting wtPFKP and SIRT2 in HEK293T cells. Using Tandem Ubiquitin binding entities (TUBE) assay (64), we observed higher ubiquitination in cells co-transfected with wtPFKP + SIRT2 plasmids vs. PFKP-plasmid alone (ubiquitination: **Figures 5F** and input: **Figure 5G**). Together, these data demonstrate that SIRT2 directly deacetylates PFKP which then promotes its ubiquitination and subsequent degradation.

**Figure 6:**
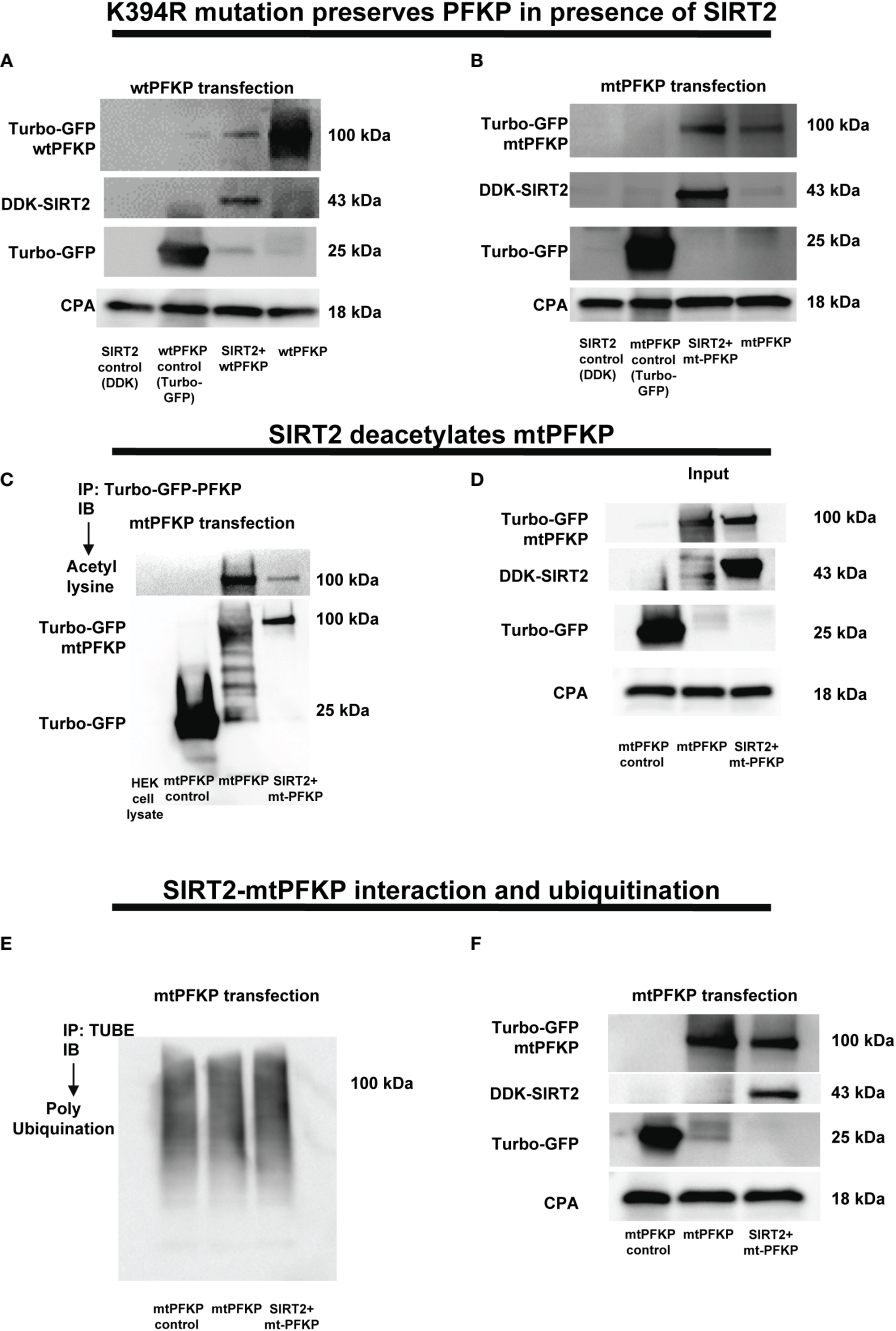
Effect of K394R mutation on PFKP. **(A, B)** HEK293T cells transfected with wtPFKP/mtPFKP in the presence or absence of SIRT2. Western blot analysis of Turbo-GFP-wtPFKP, DDK-SIRT2, turbo-GFP (control for wtPFKP plasmid transfected) and CPA. **(C)** mtPFKP transfection and IP using turbo-GFP-trap in HEK293T cells, in presence or absence of SIRT2 followed by IB analysis of acetyl lysine, turbo-GFP-mtPFKP and turbo-GFP (control for wtPFKP plasmid transfected). Pulldown with HEK293T cell lysate without transfection was used as a negative control. **(D)** Western blot analysis of turbo-GFP mtPFKP, DDK-SIRT2, turbo-GFP and CPA in whole cell lysate, used as an input for the turbo-GFP IP. **(E)** mtPFKP transfection and IP using magnetic-TUBEs in HEK293T cells, in presence or absence of SIRT2 followed by IB analysis of ubiquitination. **(F)** Western blot analysis of turbo-GFP mtPFKP, DDK-SIRT2, turbo-GFP and CPA in whole cell lysate used as input for the TUBE IP.

To elucidate the mechanisms by which the SIRT2 interaction affects the stability of PFKP, we performed a site specific mutation of mouse PFKP at K394 amino acid residue from lysine to arginine (K394R), a lysine-acetylation site known to be crucial for glycolysis (62). First, we studied the effect of SIRT2 co-transfection on stability of wtPFKP versus K394R mutant (mtPFKP) in HEK293T cells. We observed decreased wtPFKP expression in cells with wtPFKP+SIRT2 vs. wtPFKP alone (**Figure 6A**). However, the mtPFKP expression was well preserved in cells with SIRT2+mtPFKP vs. mtPFKP alone (**Figure 6B**).

To further investigate whether relatively better preservation of mtPFKP expression is due to post-translational modifications, we studied the effect of SIRT2+mtPFKP co-transfection on mtPFKP acetylation and ubiquitination. We found that the SIRT2+mtPFKP-co-transfection also led to PFKP deacetylation (immunoprecipitation: **Figure 6C** and input: **Figure 6D**), however, this deacetylation was to a lesser degree compared to wtPFKP (**Figures 5D, E**). Furthermore, the ubiquitination in

SIRT2+ mtPFKP co-transfected cells was also to a lesser degree (ubiquitination: **Figure 6E** and input: **Figure 6F**) compared to SIRT2+wtPFKP co-transfected cells (**Figures 5F, G**).

Next, we sought to answer the question of whether mtPFKP is biochemically active, by transfecting RAW 264.7 cells with mtPFKP and wtPFKP. With ethanol exposure, we observed higher basal glycolysis (**Supplementary Figure S5A**) and glycolytic capacity (supplementary figure: **Supplementary Figure S5B**) in mtPFKP- vs. wtPFKP-transfected WT-BMDM, suggesting the mutant to be enzymatically active. The green fluorescence protein (GFP) expression confirms successful transfection of plasmids (**Supplementary Figure S5C**).

These results demonstrate that mtPFKP: 1). Undergoes SIRT2 mediated deacetylation followed by ubiquitination to a lesser degree vs. wtPFKP and 2). Is enzymatically active, evidenced by its ability to participate in glycolysis. Together, we conclude that mK394 is a crucial deacetylation target of SIRT2.

### Acute ethanol-exposure decreases Light Chain3B activation

**Figure 7:**
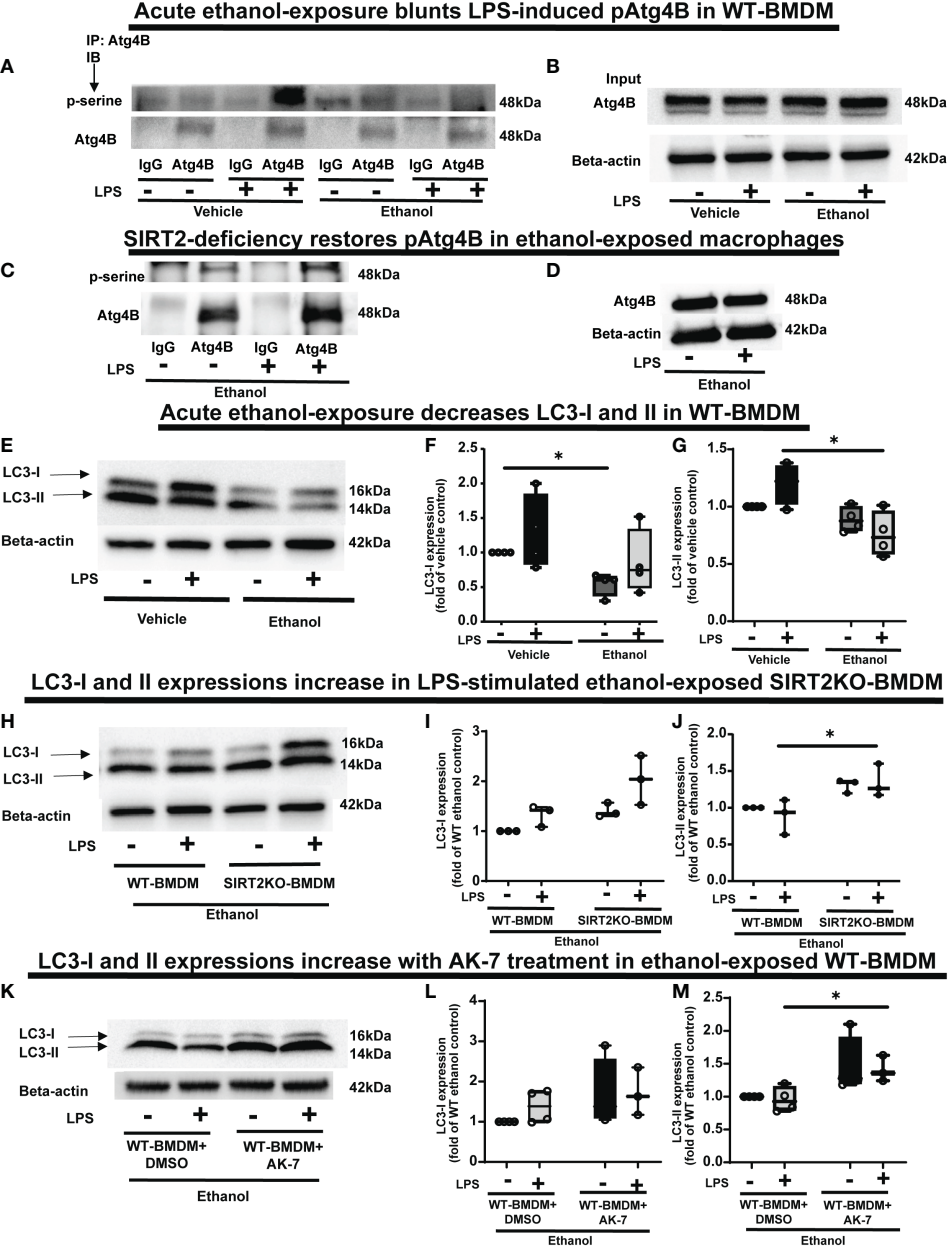
The effect of acute ethanol-exposure on pAtg4B and microtubule associated protein 1 light chain-3B (LC3) I and II expression in macrophages. **(A)** WT-BMDM were exposed to vehicle or ethanol ± LPS. Atg4B was immunoprecipitated (IP) from whole-cell lysates of WT-BMDM using an anti-Atg4B antibody followed by IB analysis of pSerine and total Atg4B. IP with IgG control antibody was used as a negative control. **(B)** Western blot analysis of total Atg4B in the whole cell lysate used as input for the Atg4B IP. **(C)** SIRT2KO-BMDM were exposed to ethanol ± LPS. IP for Atg4B from whole-cell lysates of SIRT2KO-BMDM using an anti-Atg4B antibody followed by IB analysis of pSerine and total Atg4B. IP with IgG control antibody was used as a negative control. **(D)** Western blot analysis of total Atg4B in the whole cell lysate used as input for the Atg4B IP. **(E)** LC3-I and LC3-II expression were analyzed by western blot in WT-BMDM exposed to vehicle or ethanol ± LPS. Western blot image quantification of ethanol vs. vehicle-expose WT-BMDM± LPS, showing LC3-I in **(F)** and LC3-II in **(G)**, normalized to vehicle control (Vehicle-LPS) (n = 4 blots; * *p* < 0.05). **(H)** Western blot analysis of LC3-I and II expression in Ethanol-exposed WT-BMDM and SIRT2KO-BMDM ± LPS. Western blot image quantification in Ethanol-exposed WT-BMDM and SIRT2KO-BMDM ± LPS for LC3-I in **(I)** and LC3-II in **(J)**, normalized to ethanol control (Ethanol-LPS) (n = 4 blots; * *p* < 0.05). **(K)**. Western blot analysis of LC3-I and II expression of Ethanol-exposed WT-BMDM treated with AK-7 or DMSO ± LPS. Western blot image quantification of Ethanol-exposed WT-BMDM treated with AK-7 or DMSO ± LPS for LC3-I in **(L)** and LC3-II in **(M)** normalized to ethanol control (Ethanol-LPS) (n = 3 blots; * *p* < 0.05).

PFKP acetylation is critically important for Atg4B phosphorylation, so we further investigated the effect of SIRT2-PFKP interaction on Atg4B phosphorylation and LC3 activation (43, 65, 66). Evidence suggests that LPS stimulation activates Atg4B *via* serine phosphorylation in macrophages (67). We first studied whether ethanol-exposure affects Atg4B-phosphorylation. We immunoprecipitated Atg4B in ethanol vs. Vehicle-exposed WT-BMDM ± LPS stimulation to study serine phosphorylation. As expected, we observed that Atg4B-phosphorylation increased in Vehicle-exposed (67) but not in Ethanol-exposed cells with LPS stimulation (immunoprecipitation: **Figures 7A, B**). However, Ethanol-exposed SIRT2KO-BMDM exhibited increased Atg4B-phosphorylation in response to LPS (immunoprecipitation: **Figures 7C, D**). We found total Atg4B expression to be unchanged.

Phosphorylated-Atg4B activates LC3 by priming pro-LC3 for lipidation to LC3-I and subsequently to its active form, LC3-II (65, 66). Here, we studied the effect of SIRT2-PFKP interaction and decreased Atg4B phosphorylation on LC3-activation (Step 6: **Figure 1**), in ethanol exposed cells. We found (representative image **Figure 7E**; WB image quantification: **Figures 7F, G**), that LC3-I expression decreased in ethanol vs. Vehicle-exposed WT-BMDM without LPS (54% of vehicle-LPS, a decrease by 46%, p<0.05) and with LPS (Ethanol 85% of Vehicle-LPS, decrease by 15%, p>0.05). We also found that LC3-II expression with LPS stimulation decreased in Ethanol vs. Vehicle- exposure (Ethanol 63% of Vehicle; a decrease by 37%, p<0.05). There was a strong trend towards decreased expression of LC3-II in Ethanol vs. Vehicle exposed cells without LPS (Ethanol 88% of Vehicle, a decrease by 12%, p>0.05). Ethanol-exposed cells with LPS showed a strong trend of decreased LC3-II expression vs. Vehicle-exposed cells without LPS (ethanol 76% of vehicle-exposure, a 24% decrease, p>0.05).

In contrast, we observed that both LC3-I and LC3-II expressions showed a trend towards higher expressions in Ethanol-exposed SIRT2KO-BMDM (representative image **Figure 7H**; WB image quantification: **Figures 7I, J**). Specifically, without LPS, LC3-I expression was higher in SIRT2KO vs. WT-BMDM by 40% (p>0.05). With LPS, the LC3-I expression in SIRT2KO was 100% higher than WT-BMDM without LPS (p<0.05). We observed significantly higher LC3-II expression with LPS in Ethanol-exposed in SIRT2KO vs. WT-BMDM group by 30% (p<0.05). Without LPS, we found a trend towards increase in SIRT2KO vs. WT-BMDM by 30% (p>0.05).

Similarly, both LC3-I and LC3-II expressions were higher in Ethanol-exposed and AK-7-treated WT-BMDM with LPS stimulation vs. DMSO treatment (representative image **Figure 7K**; WB image quantification: **Figures 7L, M**). Specifically, with LPS, LC3-II expression was significantly higher in AK-7 vs. DMSO treatment by 47%. Together, these data demonstrate that ethanol-exposure decreases Atg4B-phosphorylation and LC3 activation *via* SIRT2. Given the strong trends in decreased LC3 activation, we studied the effect of ethanol-exposure on LAP.

### Ethanol-exposure represses LAP *via* SIRT2-PFKP interaction

**Figure 8:**
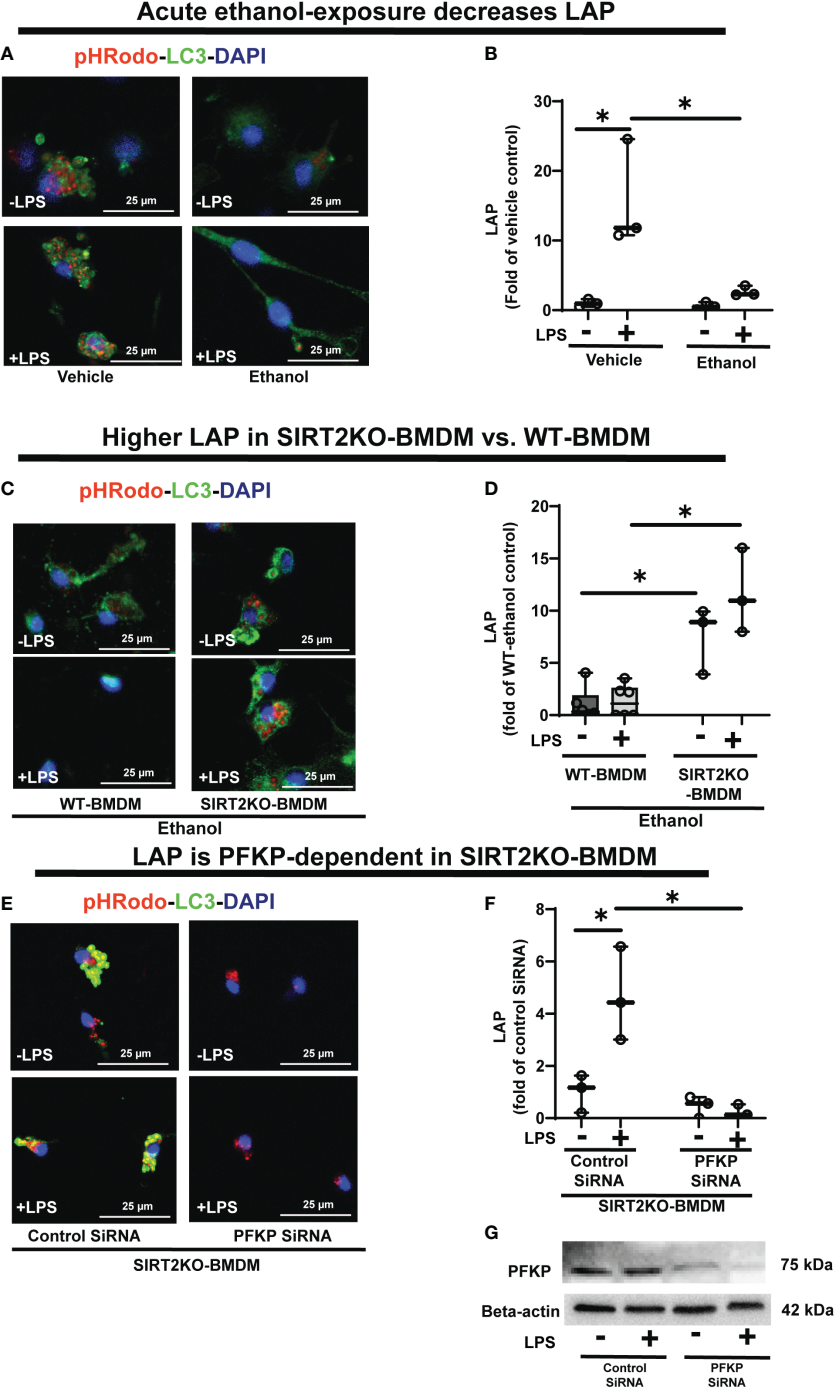
The effect of acute ethanol-exposure on LC3-associted phagocytosis in macrophages. LC3-associated phagocytosis (LAP) in WT-BMDM and SIRT2KO-BMDM, exposed to vehicle or ethanol ± LPS. **(A)** Representative images of intracellular pHrodo bioparticles during phagocytosis, co-stained for LC3. **(B)** For each image, the co-localization of intracellular pHrodo (red) and LC3 (green) were determined as LAP and divided by the total numbers of cells (nuclei). Graph represents fluorescence quantification of LAP in WT-BMDM (n=4; * p<0.05). **(C)** Representative images of LAP in WT-BMDM and SIRT2KO-BMDM exposed to ethanol ± LPS. **(D)** Fluorescence quantification of LAP in WT-BMDM and SIRT2KO-BMDM (n=5; * p<0.05). **(E)** Representative images of LAP in either control siRNA or PFKP siRNA nucleo-transfected SIRT2KO-BMDM. **(F)** Fluorescence quantification of LAP in siRNA nucleo-transfected SIRT2KO-BMDM (n=4; * p<0.05). **(G)** PFKP expression was analyzed in either control siRNA or PFKP siRNA nucleo-transfected SIRT2KO-BMDM ± LPS. PFKP protein expression was detected by western blot in whole cell lysate.

LC3-associated phagocytosis (LAP) involves incorporation of LC3-II in to LAPosome to enhance pathogen clearance (68). Since ethanol-exposure decreased LC3 activation *via* SIRT2-PFKP interaction (**Figure 7**), we wondered if that would affect LAP. First, we studied LAP in vehicle vs. Ethanol-exposed WT-BMDM (Step 7: **Figure 1**). We observed that LAP increased with LPS stimulation in Vehicle-exposed WT-BMDM by 15 fold (vehicle: +LPS/-LPS=15), but the response was profoundly muted in Ethanol-exposed BMDM to 4.3-fold increase (ethanol: +LPS/-LPS=4.3) (representative image **Figure 8A**; Image quantification: **Figure 8B**). Next, we studied Beclin-1, RUN domain-containing protein (Rubicon) and vacuolar protein sorting 34 (VPS34), proteins other than LC3 that are crucial for LAPosome formation (46). We observed a lack of differential expression between ethanol vs. Vehicle-exposed WT-BMDM ± LPS of Rubicon (representative Western Blot: **Supplementary Figure S6A**; WB quantification: **Supplementary Figure S6B**), Beclin-1 (representative Western Blot: **Supplementary Figure S6C**; WB quantification: **Supplementary Figure S6D**) and VPS34 ((representative Western Blot: **Supplementary Figure S6E**; WB quantification: **Supplementary Figure S6F**). These data demonstrate that the repression of LAP in Ethanol-exposed WT-BMDM is mainly driven by decreased LC3 activation with acute ethanol-exposure.

To investigate the role of SIRT2, we studied LAP in Ethanol-exposed SIRT2KO vs. WT-BMDM (representative image **Figure 8C**; Image quantification: **Figure 8D**). In Ethanol-exposed cells without LPS, LAP increased by 7.58-fold in SIRT2KO vs. WT-BMDM. With LPS, the LAP increased in SIRT2KO vs. WT-BMDM by 8.7 fold.

Thus, we observed that SIRT2 mutes phagocytosis, LAP and PFKP expression in WT-BMDM, while SIRT2 deficiency preserves all three with ethanol-exposure. So to further confirm the direct role of PFKP in preservation of LAP with SIRT2-deficiency, we silenced PFKP using nucleofection of PFKP siRNA in SIRT2KO-BMDM, and studied LAP (representative image **Figure 8E**; Image quantification: **Figure 8F**). We observed that the PFKP siRNA in SIRT2KO-BMDM abrogated LAP dramatically by 55% without LPS and 88% with LPS vs. control siRNA without LPS. Decreased PFKP expression with nucleofection was confirmed by western blot analysis (representative blot **Figure 8G**). These data show that the preservation of LAP in SIRT2KO-BMDM is PFKP-dependent. We also found the Atg4B-phosphorylation and LC3-I and II expressions to be decreased in PFKP siRNA vs. control siRNA in SIRT2KO-BMDM (supplementary figures: **Figures S7A, B**).

**Figure 9:**
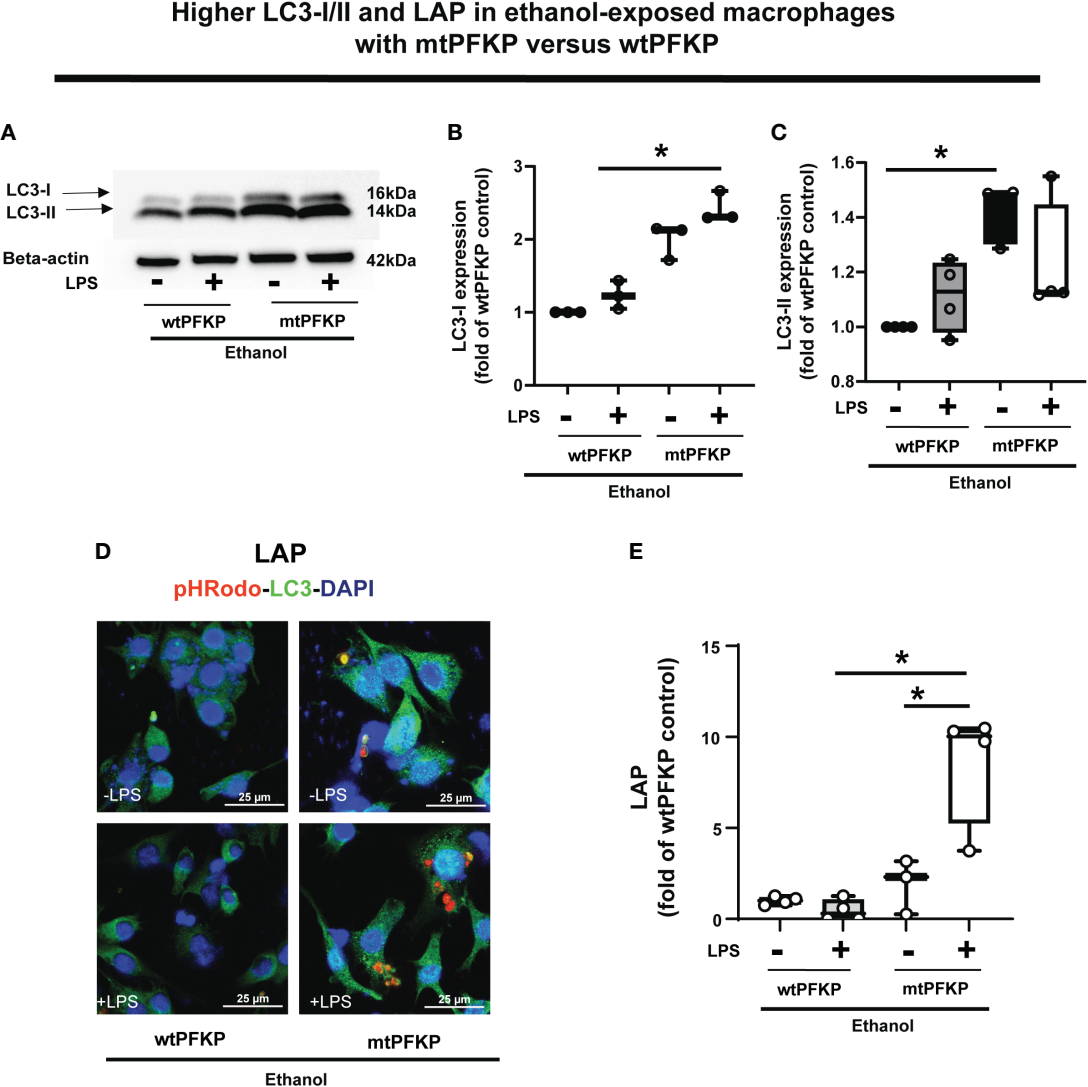
Effect of LC3-I/II and LC3-associted phagocytosis in Ethanol-exposed macrophages with wtPFKP and mtPFKP. **(A)** Western blot analysis of LC3-I and LC3-II expression in Ethanol-exposed RAW 264.7 cells with wtPFKP or mtPFKP transfection± LPS. Western blot image quantification of Ethanol-exposed RAW 264.7 cells with wtPFKP or mtPFKP transfection± LPS for LC3-I in **(B)** and LC3-II in **C** (n = 4 blots; * *p* < 0.05). **(C, D)** Representative images of intracellular pHrodo bioparticles (Red) and LC3 (green) showing LAP in Ethanol-exposed RAW264.7 cell macrophages transfected with wtPFKP or mtPFKP± LPS. **(E)** LAP quantification: For each image in “E”, the co-localization of intracellular pHrodo (red) and LC3 (green) were determined as LC3-associated phagocytosed particles and divided by the total numbers of cells (nuclei). Graph represents fluorescence quantification of LAP in WT-BMDM (n=4; * p<0.05).

**Figure 10:**
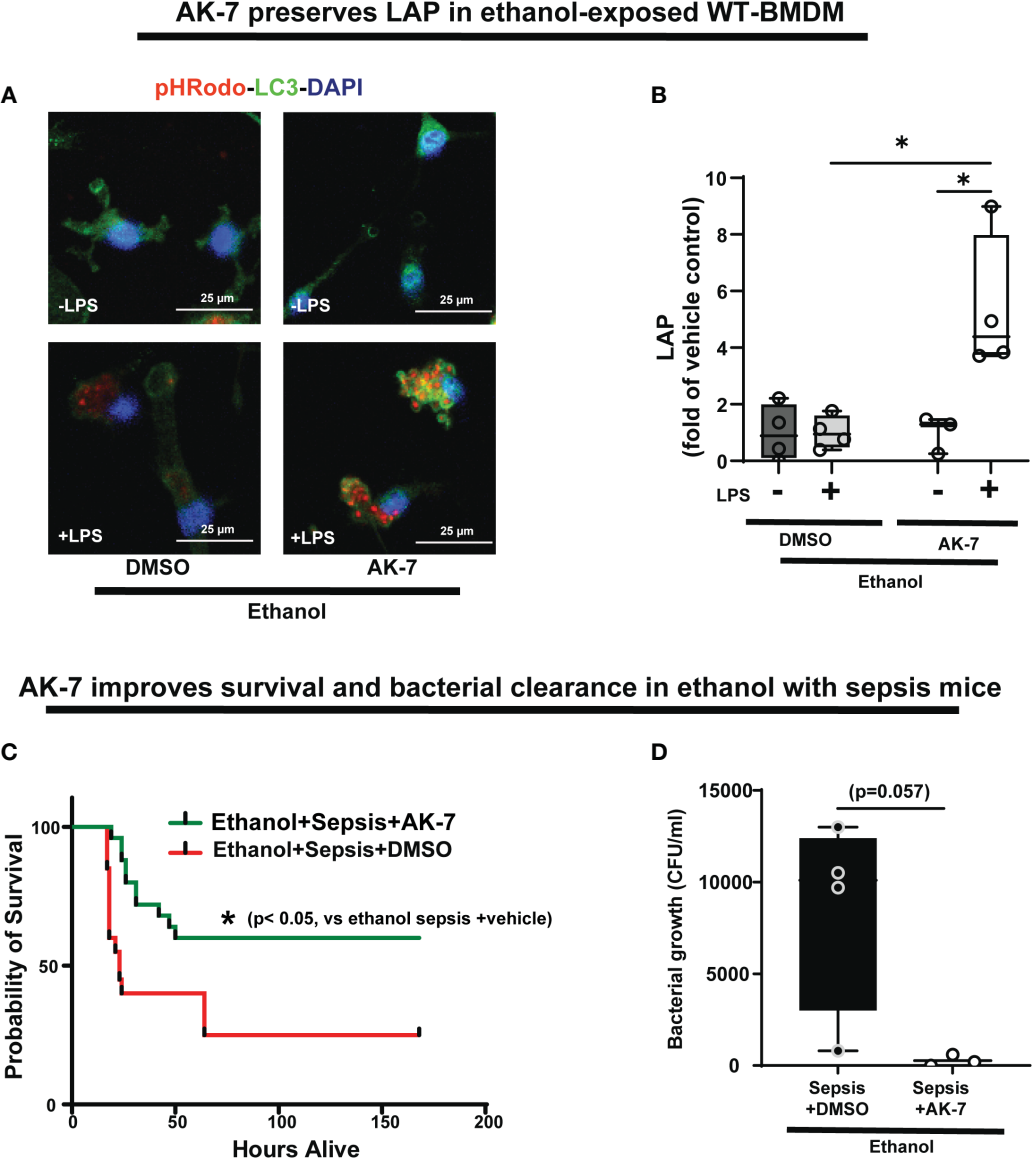
The effect of AK-7 survival in ethanol with sepsis mice and LC3-associated phagocytosis in Ethanol-exposed macrophages. **(A)** Representative images of Ethanol-exposed WT-BMDM with AK-7 or DMSO ± LPS. Staining of intracellular pHrodo bioparticles (red) and LC3 (green). **(B)** Fluorescence quantification of LAP in Ethanol-exposed WT-BMDM with AK-7 or DMSO ± LPS quantified as the co-localization of intracellular pHrodo (red) and LC3 (green) per cell (n=4; * p<0.05). **(C)** Effect of AK-7 vs. DMSO treatment on 7-day survival in ethanol-drinking WT mice with cecal slurry-induced sepsis. Kaplan-Meier survival curve shows significantly higher survival in AK-7 vs. DMSO treatment (AK-7: 60% vs. Vehicle: 25%) (n=10 each group; *p<0.05 vs ethanol sepsis + DMSO using Log-Rank test). **(D)** Peritoneal lavage from Ethanol-exposed and AK-7 or DMSO treated WT sepsis mice at 8-days post-CS injection. Bacterial colony forming units (CFU) are presented.

**Figure 11:**
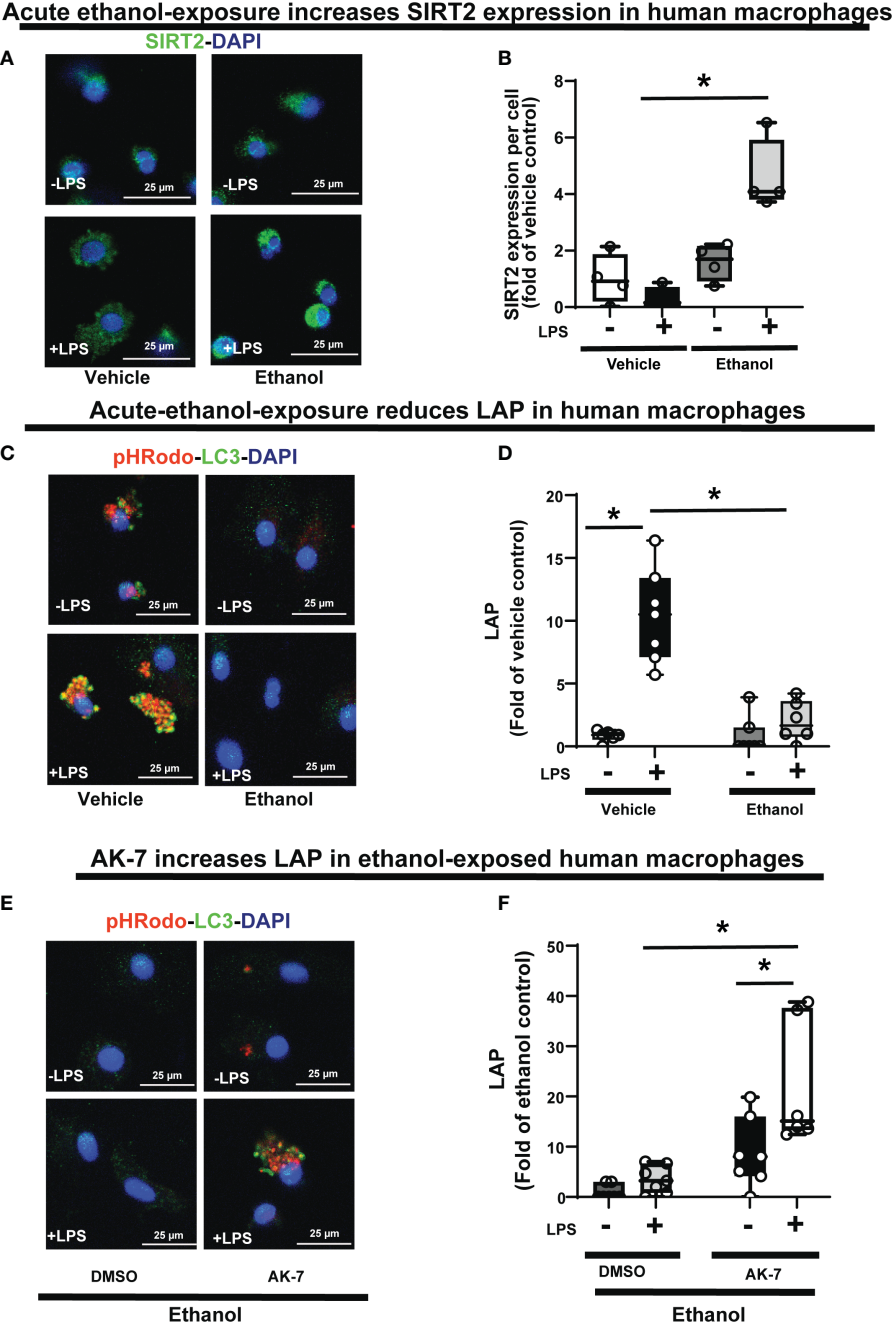
The effect of acute ethanol-exposure on SIRT2 expression and the effect of AK-7 on LC3-associated phagocytosis in Ethanol-exposed Human macrophages. Human macrophages were exposed to vehicle or ethanol ± LPS to study SIRT2 expression and LAP. SIRT2 expression was analyzed by immunostaining **(A)** Representative images of SIRT2 immunostaining in vehicle or Ethanol-exposed human macrophages ± LPS. **(B)** Fluorescence quantification of SIRT2 immunostaining in human macrophages (n=4; *p<0.05). **(C)** LAP in human macrophages with vehicle or ethanol-exposure ± LPS. Representative images of intracellular pHrodo bioparticles during phagocytosis and co-stained for LC3. **(D)** For each image, the co-localization of intracellular pHrodo (red) and LC3 (green) were determined as LAP and divided by the total numbers of nuclei. Graph represents fluorescence quantification of LAP in human macrophages (n=4; * p<0.05). **(E)** Ethanol-exposed human macrophages were co-treated with SIRT2 inhibitor AK-7 or DMSO ± LPS. Representative images of intracellular pHrodo bioparticles during phagocytosis and stained for LC3 to study LAP. **(F)** Graph represents fluorescence quantification of LAP in Ethanol-exposed human macrophages ± AK-7 ± LPS stimulation (n=4; * p<0.05).

We then examined whether the mtPFKP transfection affects LC3 expression and LAP, in Ethanol-exposed WT-BMDM (representative Western Blot: **Figure 9A** and WB quantification: **Figures 9B, C**). We observed that Ethanol-exposed and mtPFKP transfected RAW 264.7 cells showed increased LC3-I without LPS (mtPFKP-LPS/wtPFKP-LPS= 1.9 fold-increase p>0.05) and with LPS (mtPFKP+LPS/wtPFKP-LPS=2.4 fold-increase p<0.05). LC3-II expressions also increased in mtPFKP vs. wtPFKP transfection without LPS (mtPFKP-LPS/wtPFKP-LPS=1.4 fold-increase, p<0.05) and showed a trend towards increase with LPS (mtPFKP+LPS/wtPFKP-LPS=1.2 fold-increase, p>0.05). We then studied LAP in mtPFKP transfected cells (representative image: **Figure 9D**, image quantification: **Figure 9E**). Here, the LC3 was stained using Alexa Fluor 647, and artificially colored green (and *not* an indicator of turbo-GFP-tag) for congruency (with **Figures 8**, **10**, **11**). We observed that the Ethanol-exposed and mtPFKP-transfected RAW 264.7 cells showed a robust LAP vs. wtPFKP transfection in response to LPS (+LPS: mtPFKP/wtPFKP=8.5 fold-increase, p<0.05). The GFP expression in cells (**Supplementary Figure 8**) confirmed the wtPFKP and mtPFKP transfections. Thus we show that the mtPFKP is biologically active, in addition to enzymatic activity (**Supplementary Figures S5A, B**).

Together, these data demonstrate, that in Ethanol-exposed cells, increased SIRT2 represses LC3 activation and LAP *via* PFKP deacetylation at mK394.

### SIRT2 inhibitor AK-7 reverses repression of LAP with acute ethanol-exposure in macrophages, and improves survival in ethanol with sepsis mice

We then studied the effect of AK-7 on LAP in Ethanol-exposed WT-BMDM± LPS (representative image **Figure 10A**; Image quantification: **Figure 10B**). We observed that similar to SIRT2KO-BMDM (above), while DMSO-treated WT-BMDM showed repressed LAP, AK-7 treated cells showed a robust increase in LAP with LPS vs. DMSO control (AK-7+LPS/DMSO-LPS= 5.3 fold increase, p<0.05) and DMSO with LPS (AK-7+LPS/DMSO+LPS=5.24 fold-increase, p<0.05)

Previously, we reported that ethanol with sepsis decreases 7-day survival with inability to clear pathogen in mice *via* increased SIRT2 expression (9). Here, we treated Ethanol-exposed WT mice with SIRT2-specific inhibitor AK-7 (40mg/kg intraperitoneally, once) (22) and induced polymicrobial sepsis using intraperitoneal injection of cecal slurry (9). We observed significantly improved 7-day survival in AK-7- vs. DMSO-treated ethanol with sepsis mice (AK-7: 60% vs. Vehicle: 25%; p<0.005 using Log-Rank test: **Figure 10C**). In the surviving mice (day 8), we observed a strong trend towards higher bacterial growth in the peritoneal cavity (peritoneal lavage) in DMSO-treated vs. AK-7-treated mice, although this difference was not statistically significant (median CFU Sepsis+ Ethanol= 10,100 vs. Sepsis+ AK-7 = 200, p=0.057) (**Figure 10D**).

### SIRT2 represses LAP in human monocyte-derived macrophages with acute ethanol-exposure

Lastly, we confirmed the WT-BMDM findings of increased SIRT2 expression (**Graphical Abstract**: Step 2) and repression of LAP (**Graphical Abstract**: Step 7), in Ethanol-exposed human monocyte-derived macrophages ± LPS. Using immunocytochemistry, we observed a trend towards increased SIRT2 expression in Ethanol-exposed macrophages without LPS (Ethanol/Vehicle-LPS= 1.6-fold increase p>0.05) and with LPS (Ethanol+LPS/Vehicle-LPS= 4.6 fold-increase p<0.05) vs. vehicle control (representative image: **Figure 11A** and fluorescence quantification: **Figure 11B**). We confirmed these findings with Western blot (WB) analysis, which also revealed a strong trend towards increased SIRT2 (isoforms 43 kDa and 39 kDa) ± LPS (representative blot: **Supplementary Figure S9A** and WB quantification: **Supplementary Figure S9B**). We observed decreased PFKP (**Supplementary Figure S10A**) and LC3-I and II expressions (**Supplementary Figure S10B**) in Ethanol-exposed macrophages ± LPS. Similar to WT-BMDM, we observed increased LAP in Vehicle-exposed, but profoundly repressed LAP in Ethanol-exposed human macrophages with LPS (Vehicle: +LPS/-LPS= 12.6 vs. Ethanol: +LPS/-LPS= 2.3 fold-increase, p<0.05) (representative image **Figure 11C**; Image quantification: **Figure 11D**). AK-7 treated human-macrophages exhibited preserved PFKP (**Supplementary Figure S10C**), LC3-I and II (**Supplementary Figure S10D**) expressions. Lastly, we observed reversal of LAP-repression in ethanol- exposed and AK-7 vs. DMSO-treated human macrophages without LPS (-LPS: AK-7/DMSO= 10.27 fold-increase, p<0.05) and with LPS (+LPS: AK-7/DMSO=6.11 fold-increase p<0.05) (representative image **Figure 11E**; Image quantification: **Figure 11F**).

## Discussion

We report here, for the first time, that in ethanol-exposed macrophages, increased SIRT2 expression leads to PFKP-deacetylation, and that SIRT2-PFKP interaction impairs phagocytosis, mutes glycolysis, decreases Atg4B-phosphorylation and LC3 activation to repress LAP, which is crucial for bacterial clearance. Specifically, we found that SIRT2 directly interacts and deacetylates PFKP at mK394; acetylation of PFKP at mK394 is essential for its function (43). We also demonstrate that deacetylated PFKP at mK394 (hK395) is more prone to ubiquitination and degradation. Furthermore, we show that the SIRT2 deficiency (genetic deficiency or pharmacological inhibition) stabilizes PFKP expression, preserves acetylation of PFKP and glycolysis in response to LPS, Atg4B phosphorylation, LC3 activation and LAP in ethanol-exposed macrophages.

The effect of SIRT2 on phagocytosis is reported in literature, but here we demonstrate for the first time, that the SIRT2-PFKP interaction plays a critical mechanistic role in dysregulating phagocytosis, including LAP, a subset of phagocytosis crucial for pathogen degradation, in ethanol-exposure induced innate immune function (69). We have implicated SIRT2 in prolonged immunosuppression *in vivo* in obesity with sepsis mice, *via* NFκB p65 deacetylation and deactivation (22). We have also shown the effect of SIRT2 deficiency on sepsis response *in vivo* in lean mice previously (70). Here, we show the mechanism by which SIRT2 regulates immuno-metabolic response in macrophages.

Repressed LAP is known to cause sepsis-induced immunosuppression (47). LAP is a bridge between phagocytosis and autophagy (71). We observed that the ethanol-exposure dysregulates phagocytosis in macrophages, as shown in literature (32, 72, 73). A number of other proteins such as Beclin-1, Rubicon and VPS34 are crucial for LAP. We did not find the effect of ethanol exposure on the expression profiles of either of these proteins with or without LPS, suggesting that the repressed LAP observed in our studies was driven by decreased LC3 activation. Evidence suggests that Rubicon selectively enhances LAPosome formation and negatively regulates autophagy at multiple steps (44, 74, 75). While we didn’t observe differential expression, we found Rubicon to be induced in all groups of cells. Although LAP uses some of the machinery used in induction of autophagy, LAP and autophagy are two distinctly separate entities, regulated by different mechanisms (51). The distinguishing features include single membrane structure of LAP vs. double membranes of autophagosome, and the requirement of the autophagosome to be induced by pre-initiation complex, while LAP is independent of the pre-initiation complex and induced by cell surface structure (44, 53, 71).

We find that decreased expression of PFKP represses LAP *via* dysregulation of Atg4B-LC3 activation. Consistent with the published literature, we found that PFKP serves as a kinase that phosphorylates Atg4B which subsequently lipidates and activates LC3 (43). Stimulation with LPS in macrophages is known to activate Atg4B *via* phosphorylation (67). Recent evidence suggests that SIRT2 directly deacetylates and activates Atg4B to induce starvation-induced autophagy (76). In contrast however, we find that with acute ethanol-exposure induced increase in SIRT2 expression decreases Atg4B phosphorylation in WT-BMDM, which is preserved in SIRT2-deficient cells. This difference may be partly explained by biological contexts differentially regulating LC3 activation and autophagy in starvation vs. inflammatory stimuli. The biological-context-driven regulation of LC3 and autophagy need further evaluation. Also, while we have implicated SIRT2 in dysregulated autophagy with high-fat exposure, the effect of ethanol-exposure and SIRT2-PFKP interaction on autophagy in ethanol-exposed macrophages needs further evaluation (77).

Lysine acetylation of PFKP at mK394 in mice (hK395) is critical for its glycolytic function (62). We found that when co-transfected with SIRT2 plasmid, PFKP underwent degradation in HEK293T cells. Furthermore, PFKP expression was preserved in SIRT2 deficient (genetic deletion and pharmacological inhibition) macrophages (**Figures 4C-F**), suggesting SIRT2-PFKP interaction to be critical for PFKP degradation. Lysine 394 (mK394) is a crucial ubiquitination site for PFKP, in addition to acetylation. Site directed mutation of PFKP at mK394 from lysine to arginine (K394R), decreased ubiquitination and degradation of PFKP, even in the presence of SIRT2. However, the mutation did not completely abolish deacetylation of PFKP. This is somewhat expected, since we mutated one of the many acetylation sites, albeit a crucial one for its function. We contribute the partial deacetylation of mtPFKP at one or many other acetylation sites by SIRT2 and other deacetylating enzymes. Importantly however, we found, for the first time, that the mK394R mutant (mtPFKP) is enzymatically and biologically active, suggesting K394 site to be crucial for its function in regards to glycolysis and LC3 activation consistent with literature (62). We extended this finding further, to study LAP and bacterial clearance, important for sepsis survival with ethanol-exposure.

We show that the SIRT2 inhibitor AK-7 reverses repressed LAP, improves peritoneal bacterial clearance and improves survival in ethanol with sepsis mice. Thus, we speculate, that the reversal of phagocyte dysregulation with AK-7 is largely due to the LAPosome formation. Lastly, using ethanol-exposure in human monocyte-derived macrophages, we confirmed the key findings of increased SIRT2 expression, repressed LAP and reversal of LAP-repression by SIRT2 inhibition.

Acute ethanol-exposure leads to immunosuppression (38, 49, 57, 78, 79). However, chronic ethanol-exposure is shown to induce both, pro-inflammatory and anti-inflammatory phenotypes in macrophages. For example, chronic ethanol exposure shows pro-inflammatory and pro-glycolytic phenotypes in peritoneal macrophages (80). Recent evidence indicates that chronic ethanol-exposure induces pro-glycolytic phenotype in alveolar macrophages *via* HIF-1α induction (81). However, in human and experimental models, chronic ethanol-exposure induces oxidative stress in the alveolar macrophages along with suppression of phagocytosis, *via* several mechanisms such as modulation of zinc metabolism, Peroxisome Proliferator-Activated Receptor gamma (PPARγ)-regulation, AMP-activated protein kinase (AMPK) signaling amongst others (31, 82–84). Thus, the effects of ethanol on macrophages, *in vivo* and *in vitro*, are more nuanced, and several factors such as biological context, site of organ injury etc. affect macrophage behavior. While we elucidated glycolysis function here, oxidative stress and PPARγ- related modulation of macrophage function by SIRT2 in ethanol-exposed macrophages, needs further evaluation.

Our study focused on acute ethanol-exposure with immunosuppression and showed effects on glycolysis, as expected. Brain and Muscle Arnt-Like Protein-1 (Bmal1) is shown to negatively regulate macrophage polarization to M1 phenotype *via* modulation of glycolysis in experimental model of alcoholic liver disease (85). Interestingly, SIRT1-Bmal1 interactions were found to be protective of acute ethanol-exposure-induced liver injury in an experimental model (86). We have shown differential roles of SIRT1 and SIRT2 in sepsis in a biological context-dependent manner previously (22). Circadian rhythm disruption and dysregulation of CLOCK genes such as Bmal1, are reported in sepsis patients (87). The role of SIRT2-Bmal1 interactions and their effect on immune and metabolic functions of macrophages during sepsis remain to be elucidated. Similarly, a cross talk between SIRT1 and SIRT2 during acute ethanol-induced immunosuppression in ethanol with sepsis, needs evaluation.

There are several limitations to our study. Ethanol-exposure is known to impair autophagy in immune cells (88–90). We showed that the PFKP-SIRT2 interaction impairs LC3-activation, the effect of this interaction on autophagy, a critical cytoprotective pathway, needs detailed investigation with and without ethanol exposure. We pursued repressed LAP with ethanol-exposure *via* metabolic regulation. However, other molecular mechanisms responsible for repression of LAP, including transcriptional-regulation and post-translational modification of proteins responsible for phagosome formation, need further investigation (91–94). Similarly, the effect of SIRT2-PFKP interaction on cell death pathways, including necroptosis, and how they modulate autophagy, need further evaluation (95).

In conclusion, we report, for the first time, that acute ethanol-exposure induced SIRT2 interacts with PFKP, specifically targeting mouse K394 (human K395) for deacetylation leading to PFKP ubiquitination and degradation to repress Atg4B phosphorylation, LC3 activation and LAP, in macrophages. Dysregulated LAP is implicated in immunosuppression during sepsis (47, 96). We also show that SIRT2 inhibitor AK-7 reverses dysregulated phagocytosis and LAP and improves sepsis survival in mice. Thus, SIRT2 inhibition maybe a potential therapeutic option in alcohol with sepsis.

Study approvals: The animal experiments were was approved by the Institutional Animal Care and Use Committee (IACUC) at the Cleveland Clinic Lerner Research Institute (LRI). All the experiments were performed according to the NIH guidelines (ACUC approval #: 00002194, PI: Vachharajani). The human healthy volunteers without active infection and/or immunosuppression/active cancer diagnosis were consented by the Respiratory Institute Research Coordinators using an informed consent approved by Cleveland Clinic IRB (IRB # 19-132, PI: Vachharajani).

## Data availability statement

The raw data supporting the conclusions of this article will be made available by the authors, without undue reservation.

## Ethics statement

The studies involving human participants were reviewed and approved by Institutional Review Board, Cleveland Clinic, Cleveland OH 44195. The patients/participants provided their written informed consent to participate in this study. The animal study was reviewed and approved by Animal Care and Use Committee, Lerner Research Institute Cleveland Clinic, Cleveland OH 44195.

## Author contributions

Designing research studies: VV, SR, AG. Conducting experiments and acquisition of data: AG, SR, EC, CK, SA, RS, AB. Data analysis: VV, AG, SR, CK, RS. Providing reagents: LN, RS, AB. Writing and editing the manuscript: VV, AG, SR, LN, RS, AB. All authors contributed to the article and approved the submitted version.
