# Supplementary figures and images for: SIRT2-PFKP interaction dysregulates phagocytosis in macrophages with acute ethanol-exposure
Source: Front Immunol. 2023 Jan 27;13:1079962. doi: 10.3389/fimmu.2022.1079962 (PMC9972587; doi:10.3389/fimmu.2022.1079962)

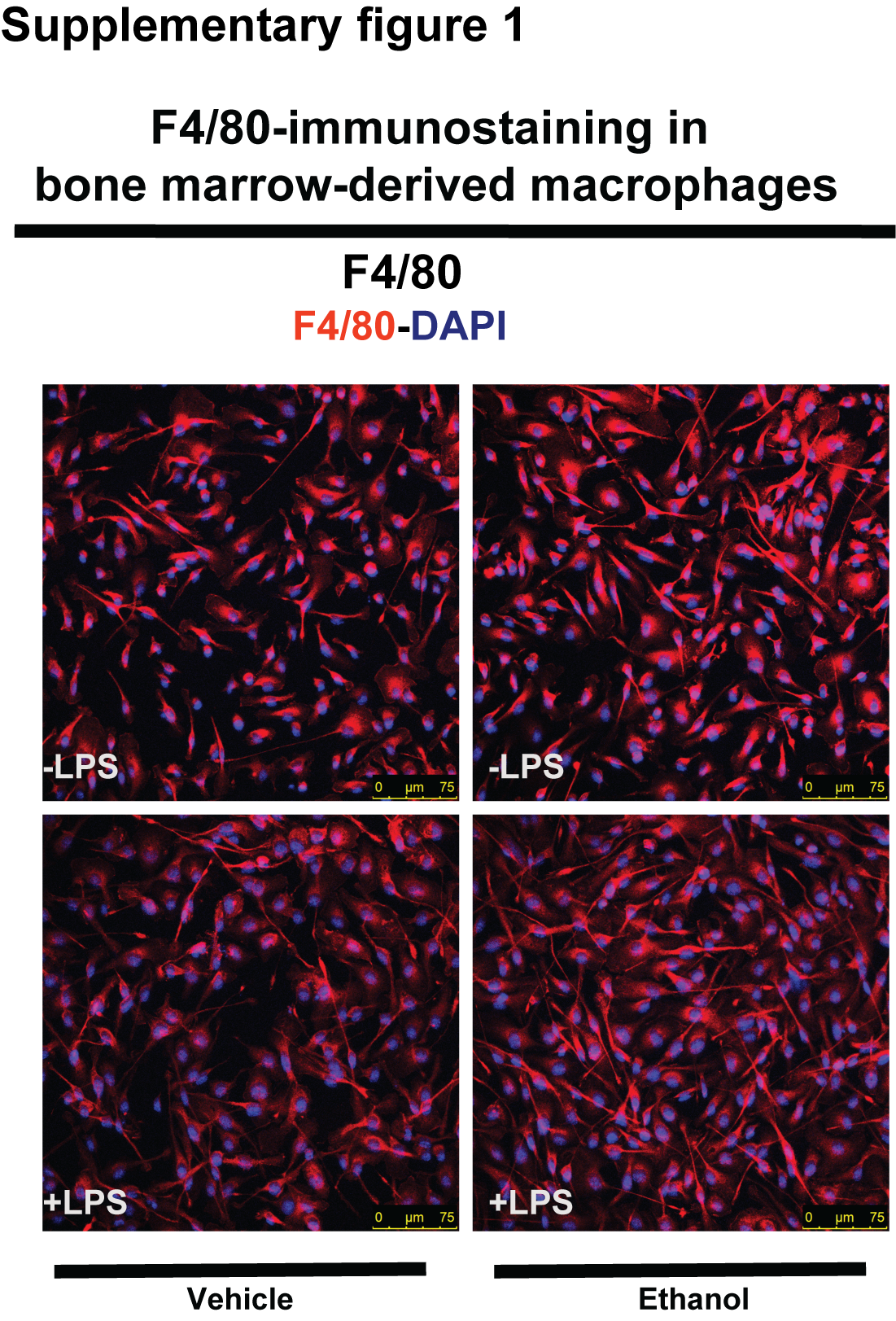

Supplement: Supplementary file 1 [file Image_1.tif]

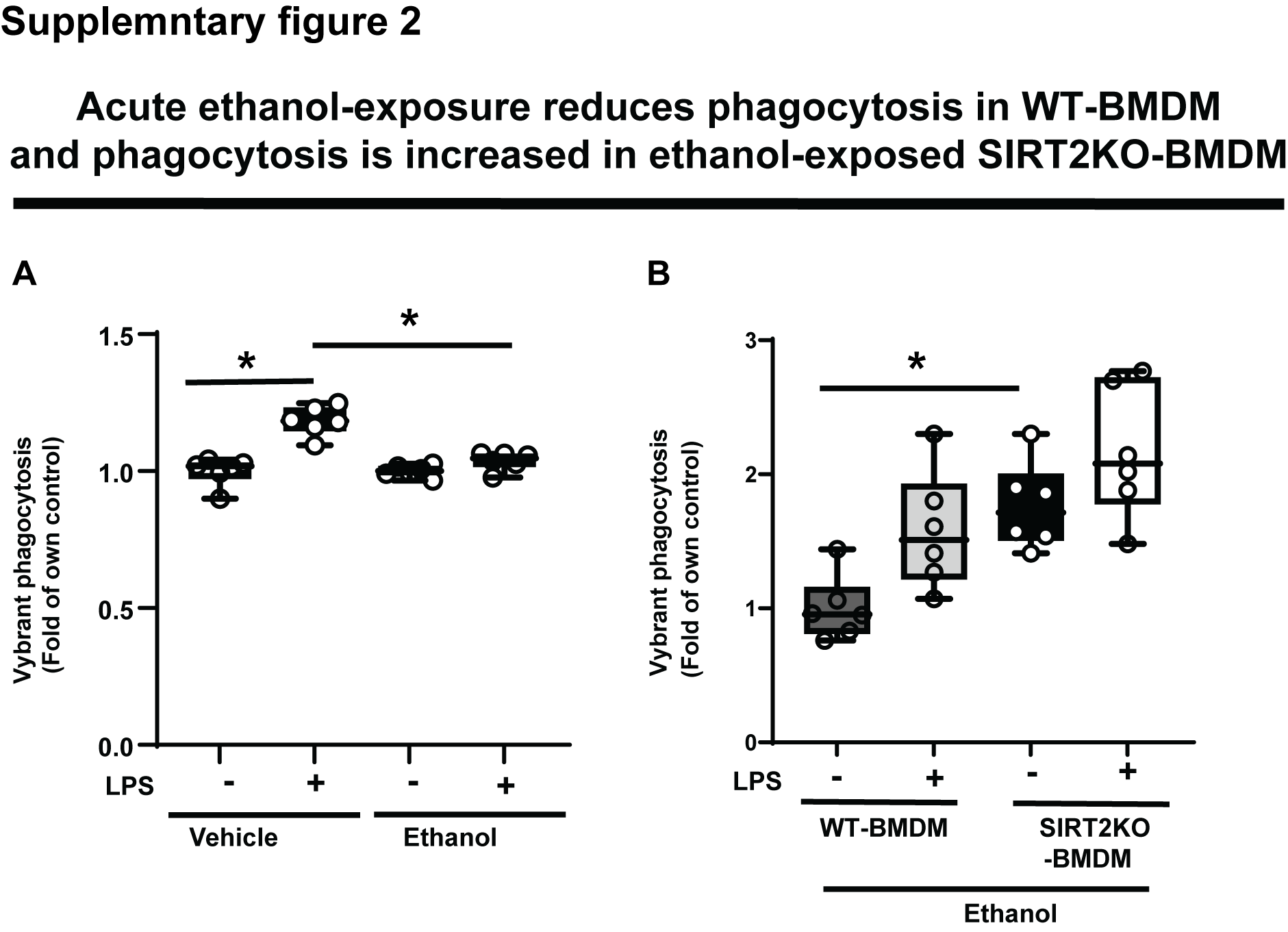

Supplement: Supplementary file 2 [file Image_2.tif]

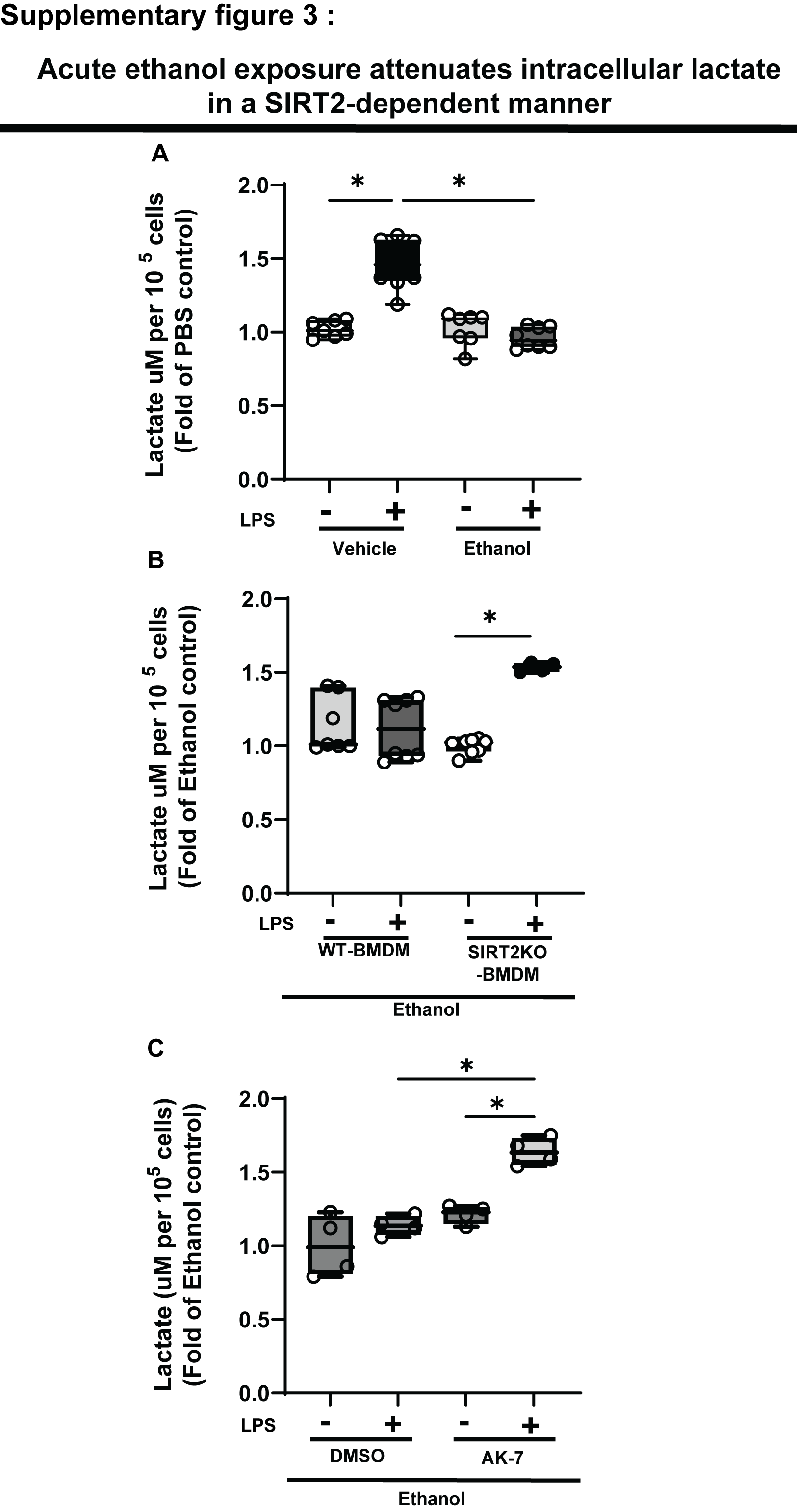

Supplement: Supplementary file 3 [file Image_3.tif]

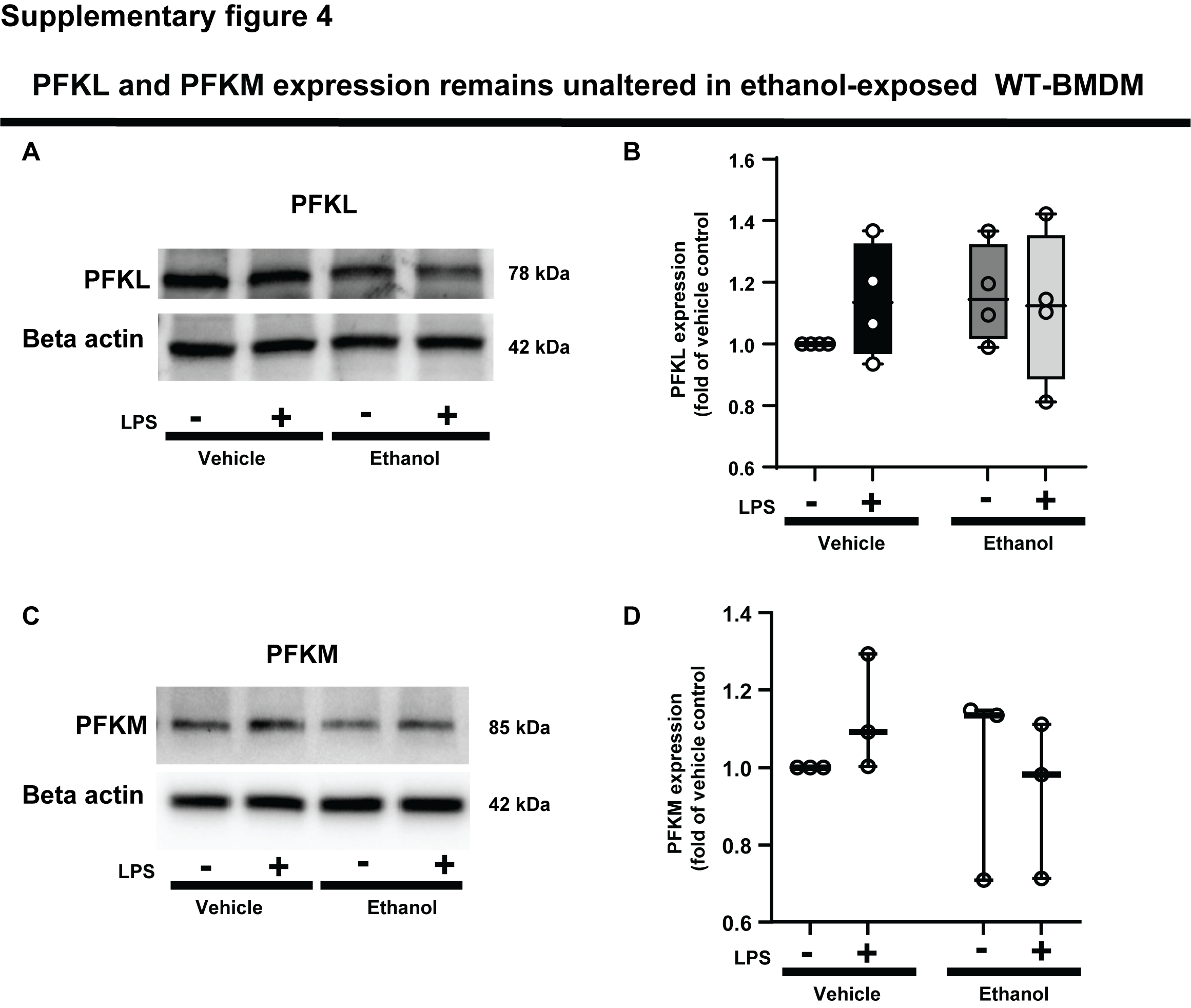

Supplement: Supplementary file 4 [file Image_4.tif]

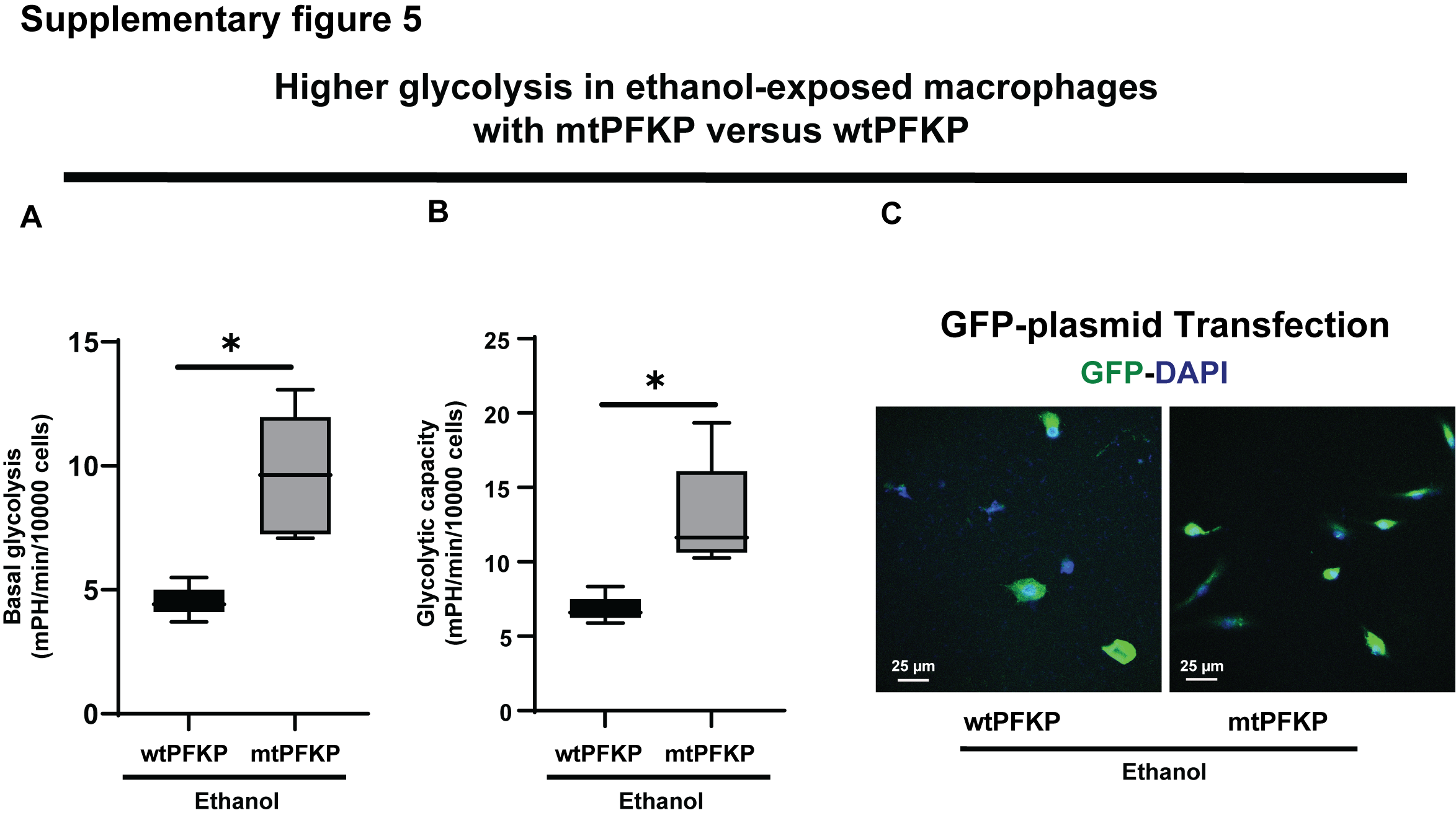

Supplement: Supplementary file 5 [file Image_5.tif]

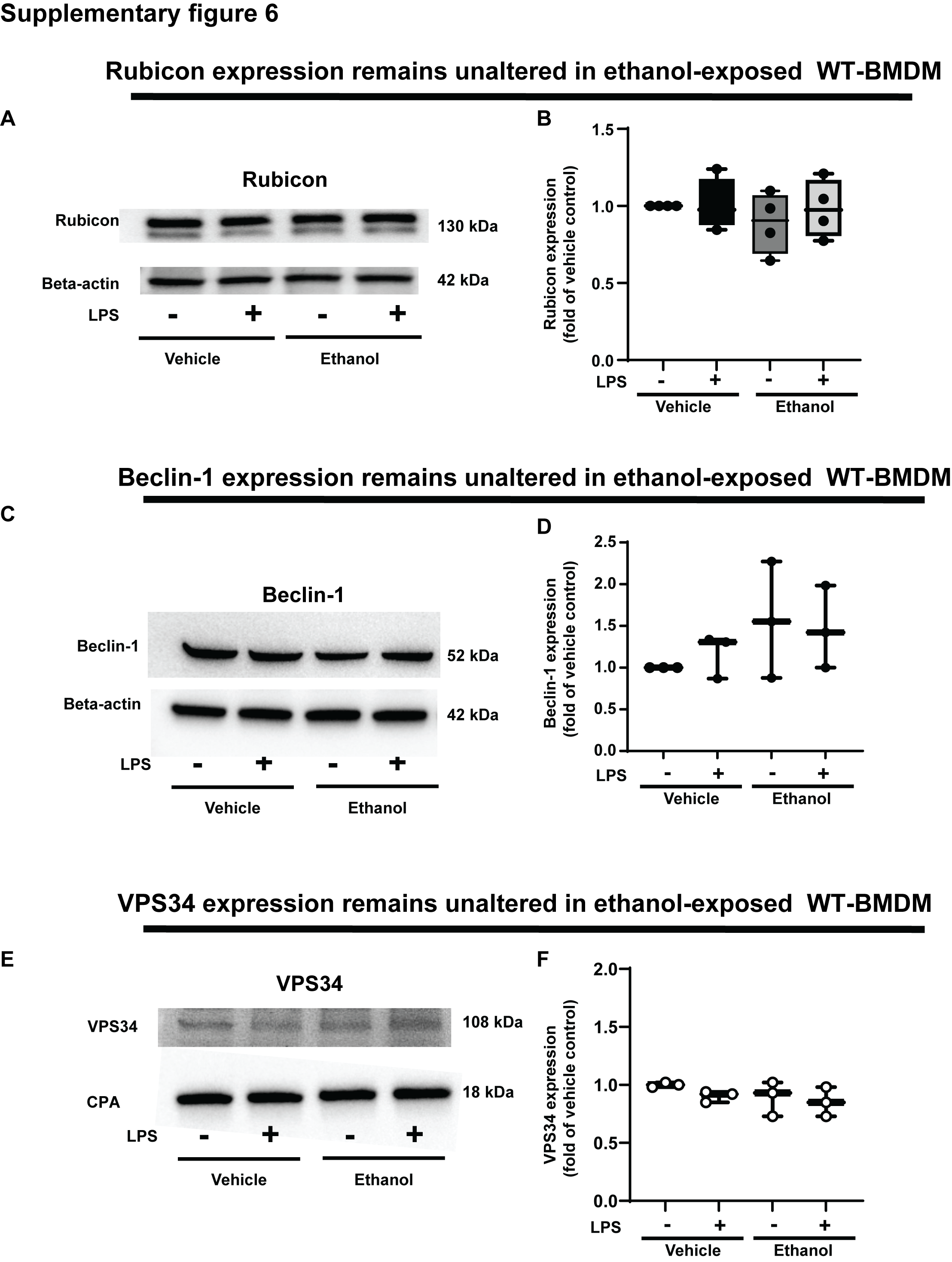

Supplement: Supplementary file 6 [file Image_6.tif]

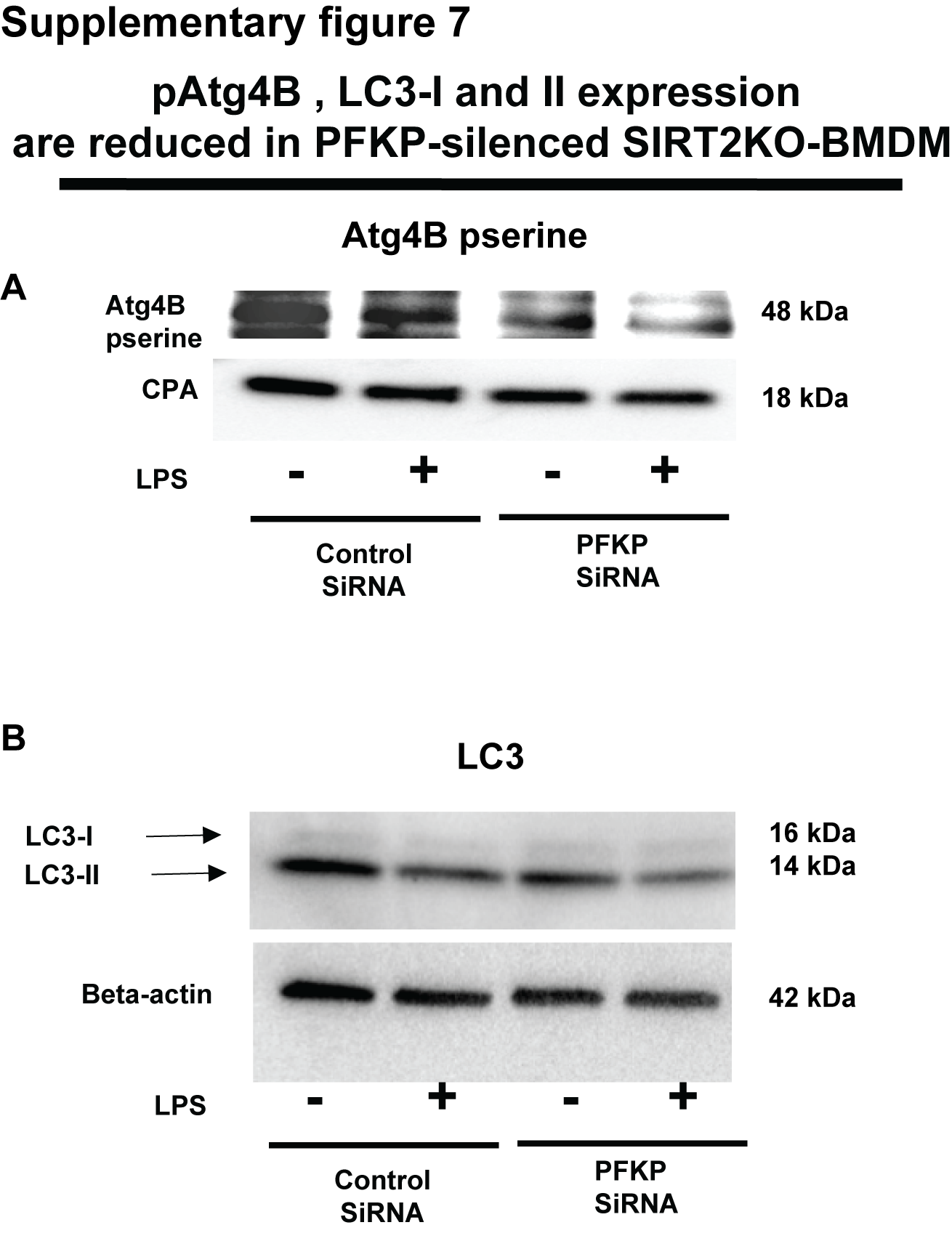

Supplement: Supplementary file 7 [file Image_7.tif]

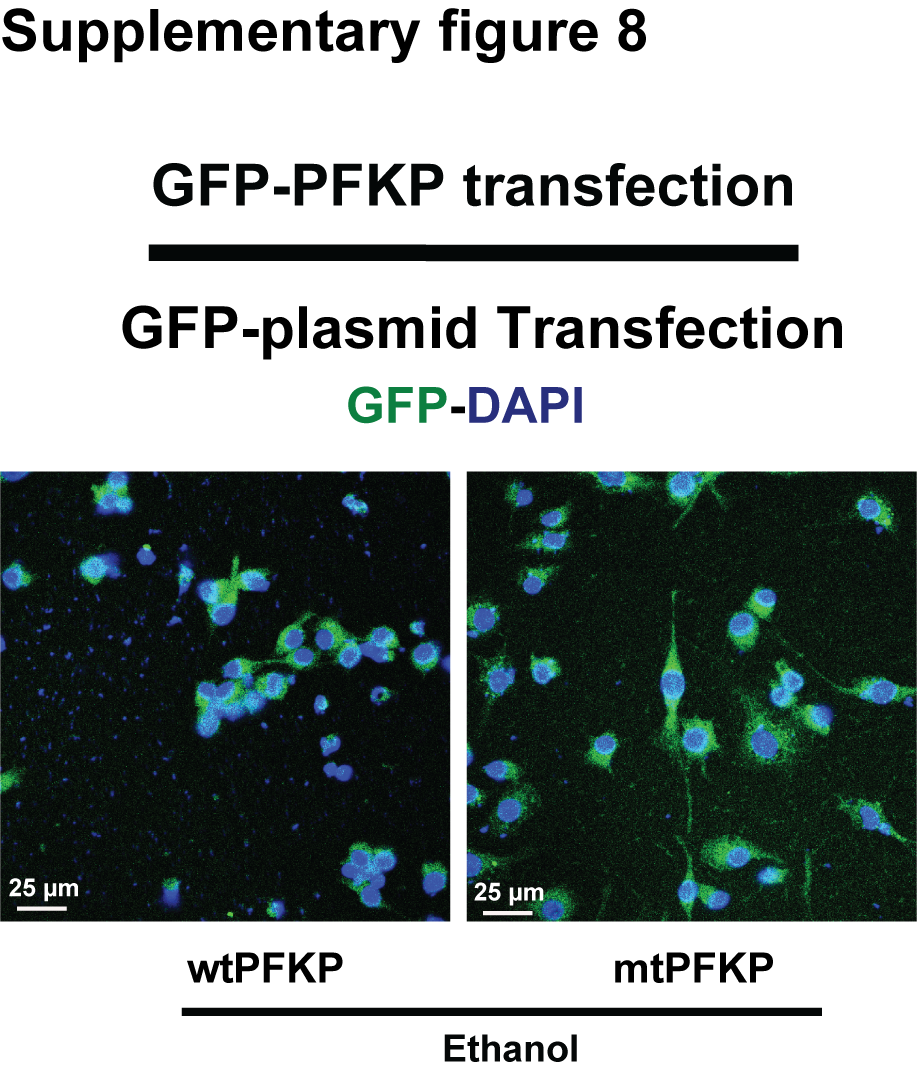

Supplement: Supplementary file 8 [file Image_8.tif]

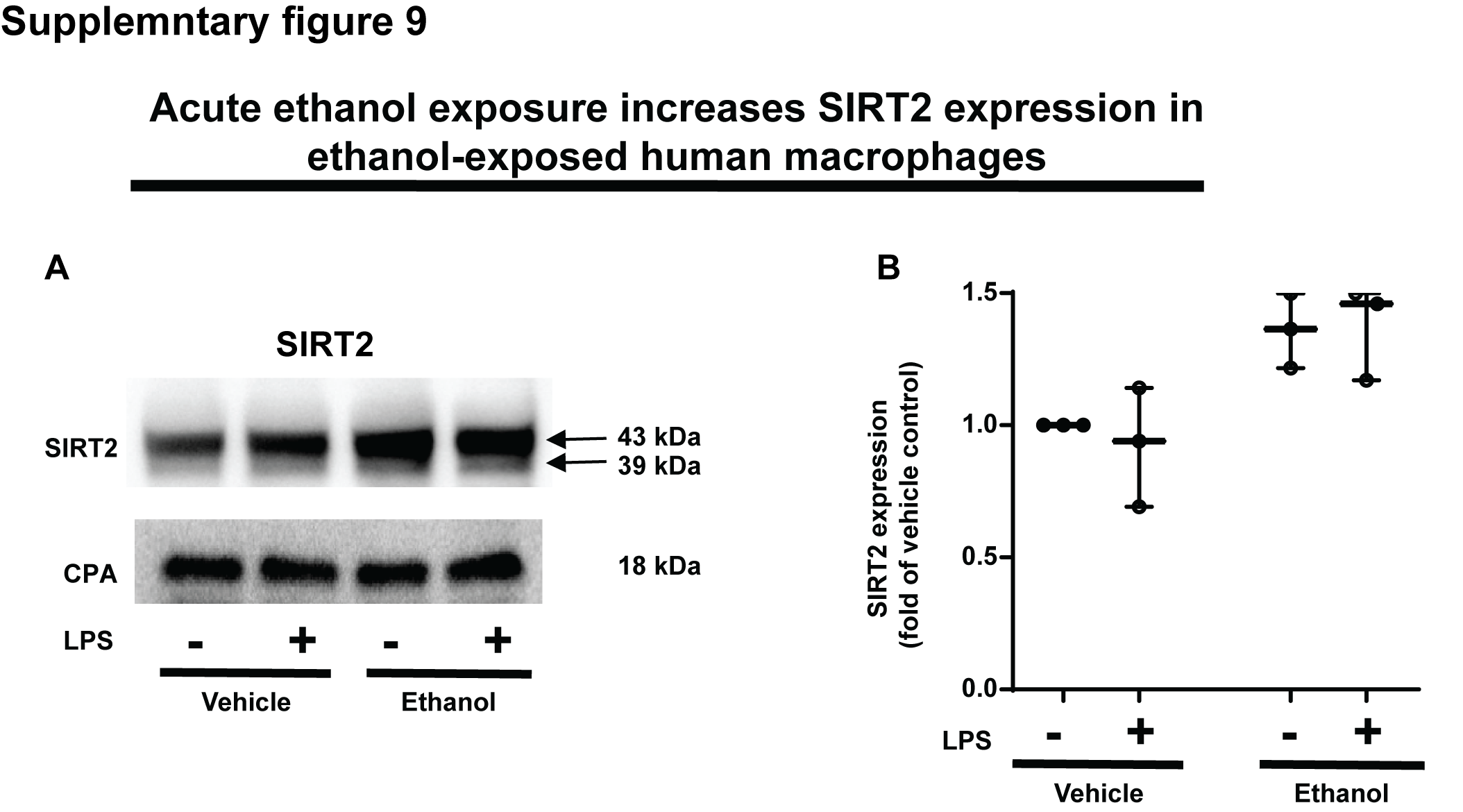

Supplement: Supplementary file 9 [file Image_9.tif]

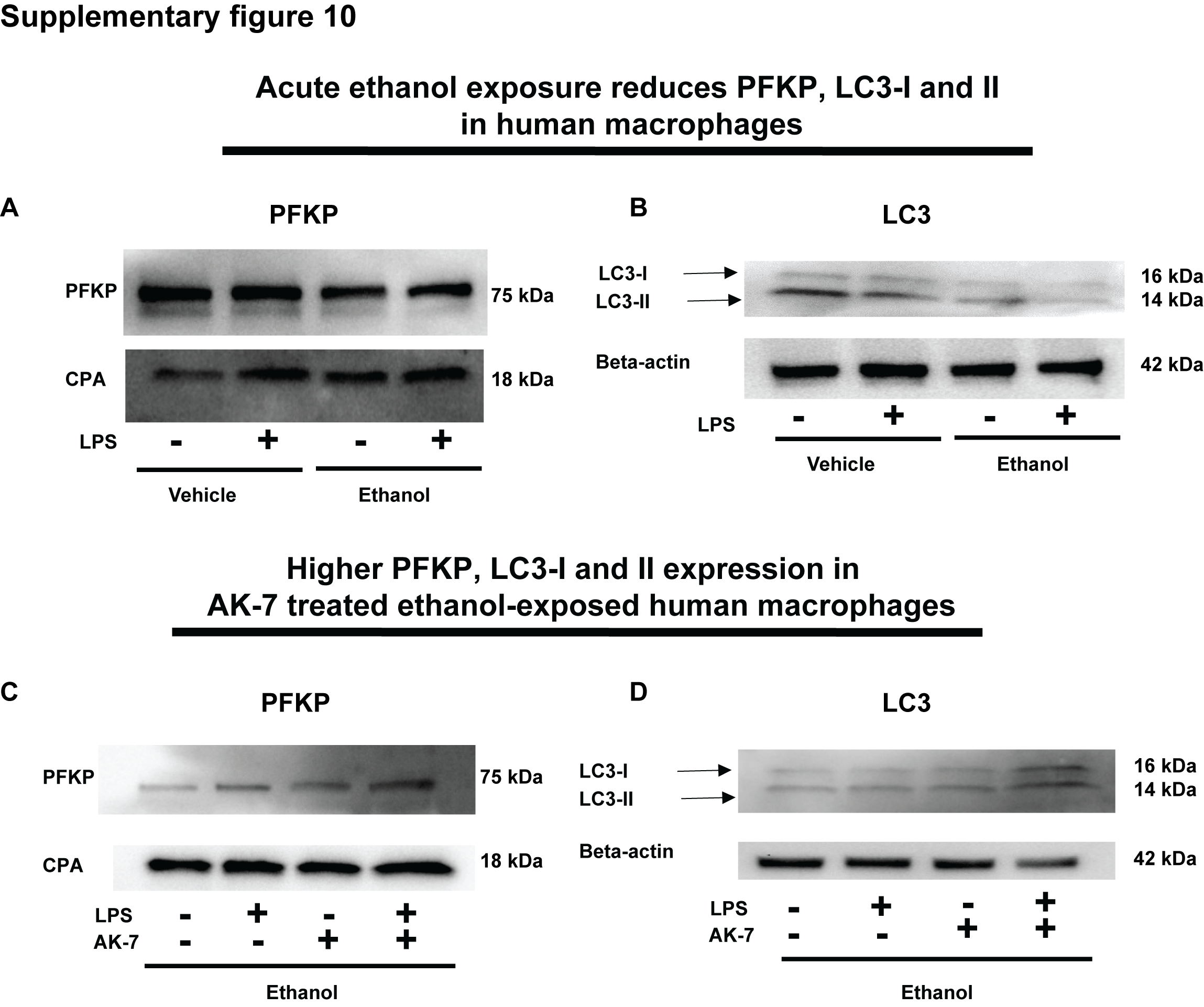

Supplement: Supplementary file 10 [file Image_10.tif]
